# Heterologous biosynthetic crosstalk with the native mansouramycin cluster in *Streptomyces albus* Del14 reveals unexpected metabolites

**DOI:** 10.1039/d5cb00235d

**Published:** 2025-12-03

**Authors:** Marc Stierhof, Liliya Horbal, Patrick Oberhäuser, Anja Palusczak, Peyton Cox, Maria Lopatniuk, Christopher Ruf, Josef Zapp, Andriy Luzhetskyy

**Affiliations:** a Helmholtz Institute for Pharmaceutical Research Saarland (HIPS) Germany andriy.luzhetskyy@uni-saarland.de; b Saarland University, Pharmaceutical Biotechnology Campus C2 3 66123 Saarbrucken Germany; c Saarland University, Pharmaceutical Biology Campus C2 3 66123 Saarbrucken Germany

## Abstract

*Streptomyces albus* J1074 (now *S. albidoflavus* J1074) is a widely used heterologous host for natural product discovery due to its capacity to express biosynthetic gene clusters (BGCs) from diverse organisms. A derivative of this strain, *S. albus* Del14, enhances heterologous expression by reducing background metabolite production enabling the identification of the previously hidden BGC responsible for producing mansouramycins. In this study, we demonstrate the biosynthetic crosstalk between the native mansouramycin BGC in *S. albus* Del14 and introduced BGCs from three different organisms results in the production of novel compounds, some featuring rare and complex chemical scaffolds. These include malevonin, which combines NRPS- and mansouramycin-derived building blocks forming a fluorene scaffold, as well as 5′-chloromansouramycin D, a halogenated derivative of mansouramycin D. Additionally, we identified mansevorone, a compound structurally similar to mansouramycin D but utilizing a different tryptophan-derived C7 precursor. This precursor likely arises from the activation of native genes in the host *S. albus* Del14, triggered by SARP regulators present on the introduced BGC. These findings highlight the evolutionary significance of BGC interactions and underscore their potential as a powerful tool for discovering novel natural products, providing insights that could inform innovative strategies in biosynthetic engineering and the guided evolution of new bioactive compounds.

## Abstract 2

Biosynthetic interactions between *Streptomyces albus* Del14 and heterologous gene clusters lead to the production of new metabolites.

## Graphical abstract

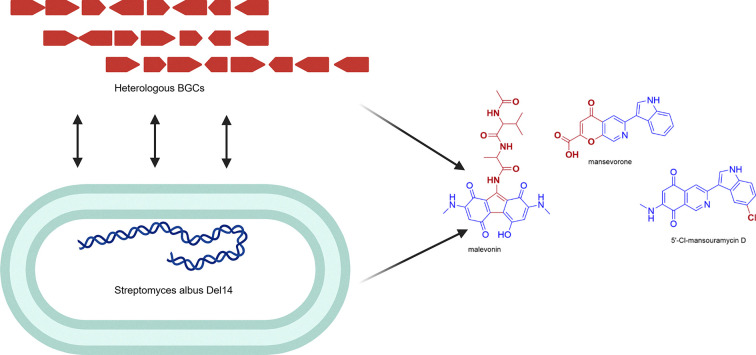

## Introduction

Over the last two decades *Streptomyces albus* J1047 has successfully been used as a heterologous expression host to study and discover a large variety of natural products.^1–6^ To facilitate access to these metabolites and optimize expression conditions, a chassis strain was developed based on *S. albus* J1047, namely *S. albus* Del14. Deletion of 15 biosynthetic gene clusters (BGCs) in *S. albus* Del14 led to a reduced metabolic background and higher production titers, which enabled the successful expression of previously inaccessible natural products.^7^

**Fig. 1:**
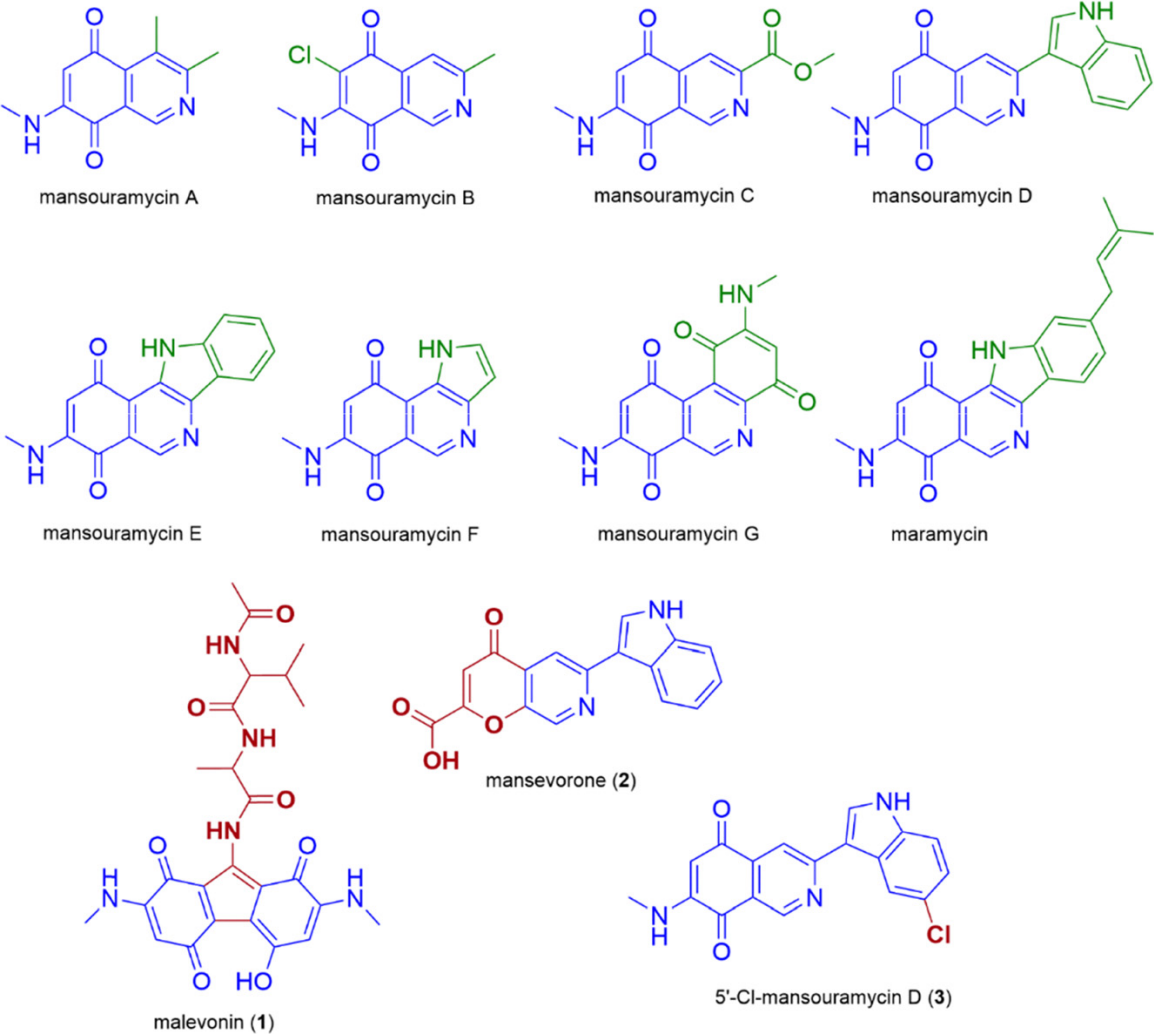
Structures of all known mansouramycins, maramycin and the newly discovered malevonin (1), mansevorone (2) and 5′-Cl-mansouramycin (3).

The simplified production profile of *S. albus* Del14 resulted in the appearance of new compounds later identified as mansouramycin A and D. Due to their unknown biosynthesis, the corresponding biosynthetic gene cluster was not annotated by genomic search tools like antiSMASH and only showed production when the rest of the metabolic background was cleared. In a study by Hui *et al.*, it was discovered that the cluster utilized tryptophan (Trp) as a building block, which draw the focus on Trp-related genes. This finding subsequently enabled the identification of the BGC responsible for mansouramycin biosynthesis and the elucidation of its biosynthetic pathway until the C7 precursor, while the final biosynthetic mechanism leading to mansouramycins A–G remains unknown (Fig. 1).^8–10^ In the course of this study, the mansouramycin BGC-deficient strain *Streptomyces albus* Del15 was generated. However, it exhibited reduced fitness compared to *S. albus* Del14. Therefore, *S. albus* Del14 was selected as the primary host for our heterologous expression pipeline.^11^

Most of the BGCs successfully expressed in *S. albus* Del14 produced compounds that were predicted using *in silico* tools. However, in some cases, the structure of the newly identified metabolites showed significant differences to the predicted one and seemed to incorporate structural elements from mansouramycin D, resulting in a great variety of new compounds. We identified three of such compounds, including malevonin (1) with a substituted fluorene scaffold, mansevorone (2) with an azachromone scaffold and 5′-Cl-mansourmaycin D (3), a chlorinated variant of mansouramycin D. By conducting feeding studies, knockout experiments, and heterologous production in the mansouramycin BGC deficient strain *S. albus* Del15, we demonstrated that mansouramycin biosynthesis interacts with the foreign biosynthetic genes, leading to a variety of different new compounds. This observation points to the flexibility of the mansouramycin biosynthetic pathway, which permits crosstalk with heterologously introduced genes. Such bacterial crosstalk has been exploited in previous studies and has led to the production of hybrid compounds, including hybrubins and sipanmycin analogues.^12–14^

In this study, however, compounds arising from biosynthetic crosstalk with the mansouramycin pathway were not the primary focus; rather, their emergence was an unexpected yet intriguing finding. Because horizontal gene transfer is a major driver of natural product diversity in *Streptomyces.* As a consequence, the potential for cluster crosstalk must be also taken into account when employing *Streptomyces* as a host for heterologous expression.

A deeper understanding of the mechanisms underlying mansouramycin-related crosstalk could ultimately be harnessed to design new molecular scaffolds once these interactions are fully elucidated.

## Results and discussion

### Heterologous production of malevonin

**Fig. 2:**
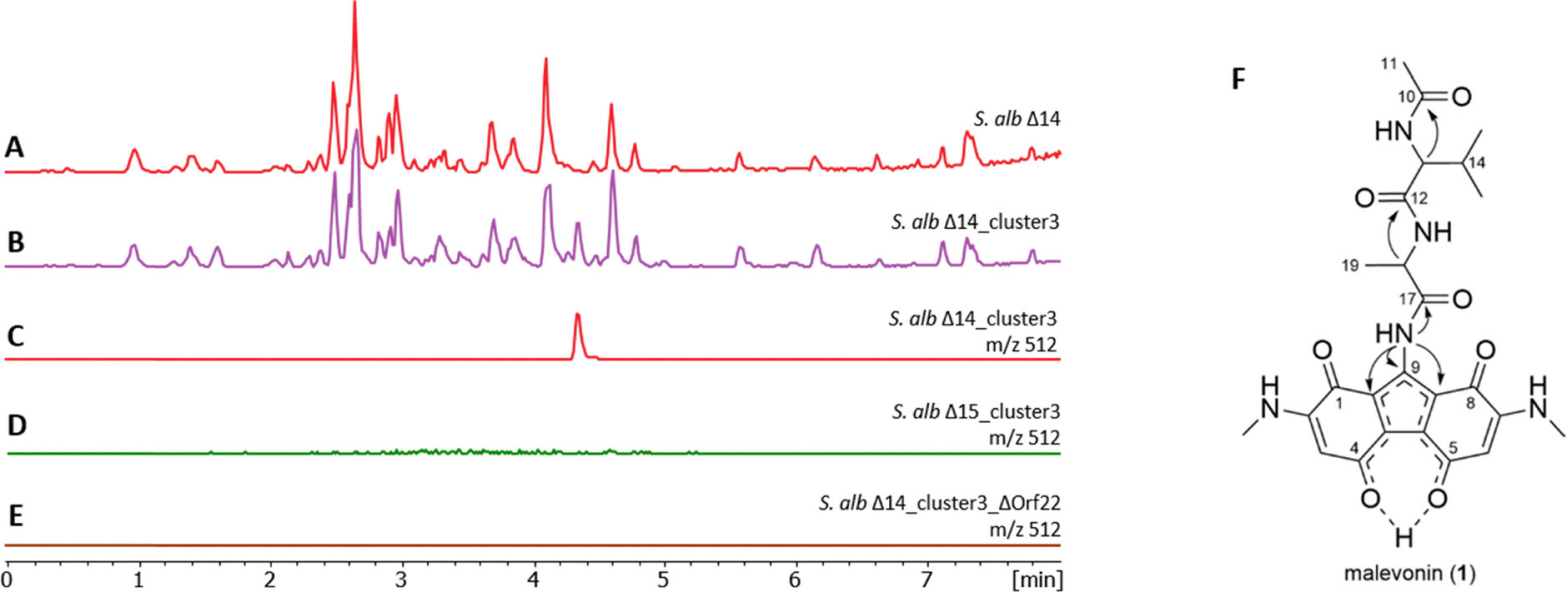
Base peak chromatogram of the *S. albus* Del14 chassis strain (A), heterologous expression of the cluster 3 in *S. albus* Del14 (B) showing production of malevonin (C), abolished malevonin production in the mansouramycin deficient strain *S. albus* Del15_cluster3 and (D) abolished malevonin production in the NRPS deletion mutant *S. albus* Del14_cluster3_delOrf22 (E). The mesomeric structure of malevonin and key HMBC correlations (
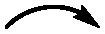
) are shown in F.

In our heterologous expression pipeline we constantly express biosynthetic gene clusters from various organisms in our *in house* chassis strains *S. lividans* Del8 and *S. albus* Del14.^15–21^ Mutant strains are cultivated in SG and/or DNPM medium, metabolites are extracted by butanol and/or ethyl acetate and analysed by LC-HRMS. New peaks are identified by dereplication through comparison with the Dictionary of Natural Products and the Natural Product Atlas, allowing differentiation of known compounds from potentially novel ones.^22,23^ One of the clusters from our pipeline, cluster 3, originated from cosmid library created for *S. kitasatoensis* (GenBank acc. nr. PV759347.1). Cluster 3 was selected based on 54% similarity to vazabitide A biosynthetic genes by antiSMASH.^24^ The cluster showed differences in the NRPS core genes and some additional biosynthetic genes that suggested a new compound different from vazabitide A. Heterologous expression of the cluster 3 in our second chassis strain *S. lividans* Del8 did not lead to any new peaks. However, in *S. albus* Del14 we observed new ions, including one at *m*/*z* = 512.2145 corresponding to the proton adduct [M + H]^+^ of a major compound, and we also observed green coloration of the bacterial culture (Fig. S1, S2A and Fig. 2(A)–(C)). The compound was rather insoluble, therefore, it was possible to purify it by washing the bacterial butanol extract with hexane and methanol resulting in 6 mg of the compound. The pure compound showed pH-related changes in the UV absorption bands at high and low pH, indicating a large conjugated structure with pH-sensitive groups (Fig. S2B).

The molecular formula was calculated as C_25_H_29_N_5_O_7_ with 14 degrees of unsaturation based on *m*/*z* = 512.2145 [M + H]^+^ Δ − 0.9 ppm (calc. *m*/*z* = 512.2140). Due to its low solubility, the compound was dissolved in DMSO-d_6_ to saturation (*ca.* 2 mg in 300 µL) and the structure was determined by NMR (Table S6 and Fig. S3–S9). Analysis of ^1^H, ^13^C and edited-HSQC spectra revealed five methine, six methyl and fourteen quaternary carbons. Four proton signals above 7 ppm did not show correlations to carbon signals and were determined as NH groups. COSY spectra revealed two spin systems, which were assigned to alanine (Ala) and valine (Val). Long-range HMBC correlations showed that the two amino acids form a dipeptide with an N-terminal acetyl group (Ac) resulting in Ac–Val–Ala. The calculated remaining molecular formula C_15_H_12_N_3_O_4_ did not match the observed signals from the 1D experiment that indicated a molecular formula of C_8_H_6_N_2_O_2_, suggesting a symmetrical moiety. This was confirmed by comparing the integral values in the ^1^H-NMR, where 9-NH showed a value of 1, while 3/6-CH, 2/7-NH, and 2/7-NMe displayed twofold integral values. Assignment of all peaks with twofold integral values led to two 2-(methylamino)-1,4-benzoquinone (MAB), a structural feature of mansouramycin. The HMBC correlation from 9-NH to 1a/8a-C, along with a weak correlation to 9-C, suggested that 9-C is adjacent to both 9-NH and 1a/8a-C, thereby linking the two MAB moieties (Fig. 2(F)). Based on the established structure, the final connection needed to achieve the required 14 degrees of unsaturation is between the quaternary carbons 4a-C and 5a-C resulting in a 5-hydroxy-bis(methylamino)-1*H*-fluorene-1,4,8-trione. The symmetry is achieved by the enol hydroxyl proton that forms hydrogen bonds between the oxygen atoms at position 4 and 5, resulting in a mesomeric diketonate (Fig. 2(F)). In this configuration, the proton is significantly deshielded, resulting in a downfield-shifted signal at 18.47 ppm (Fig. S4). A review of the literature revealed a structurally similar compound, namely hipposudoric acid,^25^ which exhibited a comparable chemical shift of 16.05 ppm for the deshielded proton, supporting our observations. The absolute configuration of the amino acid residues was determined by Marfey's method, which resulted in l-Ala and l-Val (Fig. S10), and the compound was named malevonin (1).^26^

### Cluster 3 enzymes utilizes the tryptophan-derived C7 precursor

Malevonin differs significantly from the expected cluster product, which was anticipated to be a vazabitide A derivative. Instead, it features a substituted fluorene scaffold incorporating two MAB moieties—structural elements derived from mansouramycin D—suggesting an interaction between the native mansouramycin biosynthetic cluster and the introduced cluster 3. To verify this, cluster 3 was expressed in the mansouramycin BGC deficient strain *S. albus* Del15. Subsequent analysis of the metabolic profile of the *S. albus* Del15_cluster3 mutant did not show production of malevonin or other new compounds (Fig. 2(D)). This indicates that cluster 3 requires structural elements generated by the native mansouramycin BGC to form malevonin.

As demonstrated by Hui *et al.*, the mansouramycin BGC uses tryptophan (Trp) as a building block for the Trp-derived C7 precursor 5-(methylamino)-3,6-dioxocyclohexa-1,4-diene-1-carboxamide (5).^11^

**Fig. 3:**
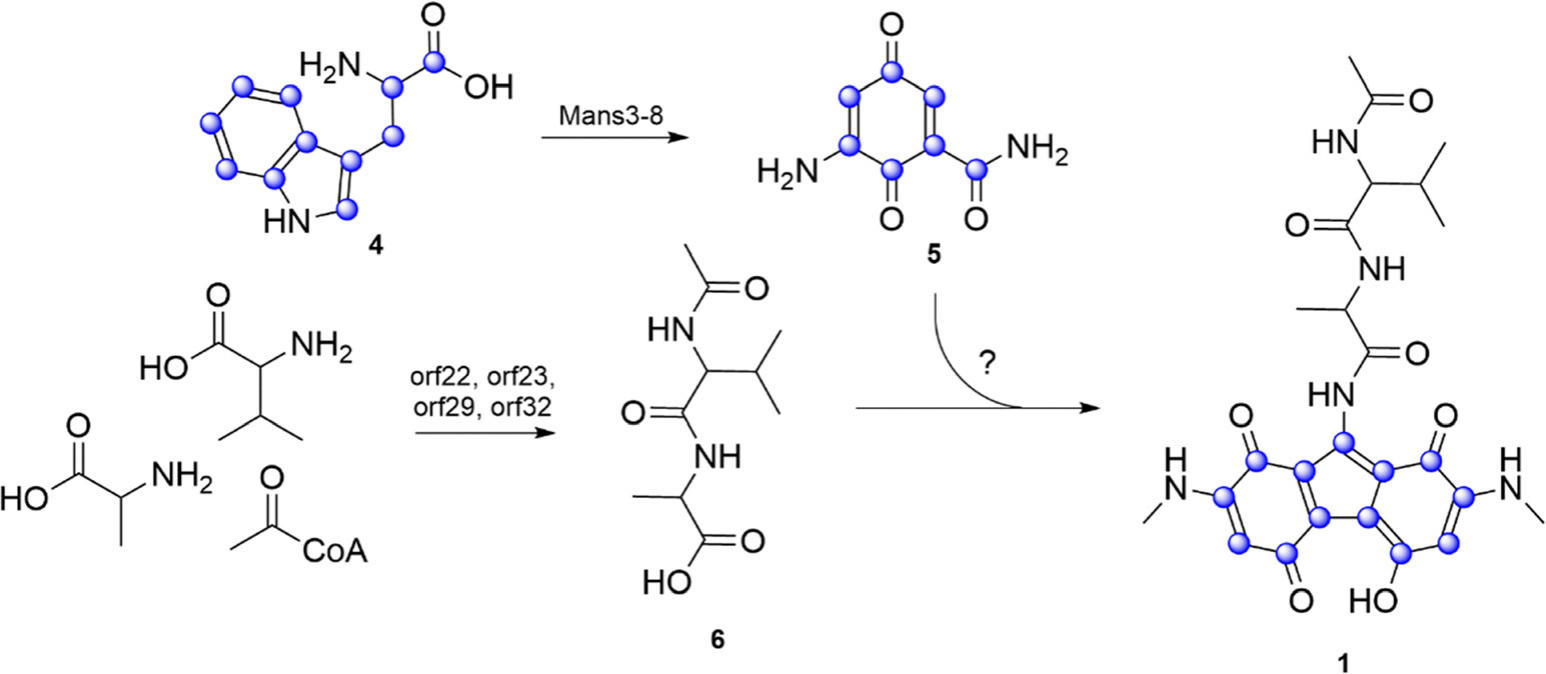
Proposed biosynthetic pathway of malevonin also showing the labeling pattern when ^13^C_11_ Trp (4) is fed (blue dots).

To determine if the fluorene core in malevonin is constructed from the C7 fragment, *S. albus* Del14_cluster3 was fed with ^13^C_11_ labelled Trp (4) and the culture extracts were analysed by LC–MS. The feeding was done in rich medium, providing both labelled and unlabelled Trp as building blocks. Subsequent analysis revealed the unlabelled malevonin mass peak and masses differing by +6, +7 and +13 Da, indicating an incorporation of a C7 and a C6 fragment, both originating from 5 (Fig. S11 and Fig. 3). Concluding from that, the fluorene core is likely synthesized from two moieties of the Trp-derived C7 precursor (5).

The peptide moiety of malevonin was hypothesized to originate from cluster 3, which was analysed through *in silico* gene analysis. The chromosomal fragment of cluster 3 consists of 53 genes, including *orf6-orf36* which contain the genes *vzb0*, *vzb3* and *vzb5-28* from vazabitide biosynthesis (Table S5).^24^ The presence of three NRPS genes (*orf22*, *orf23* and *orf32*) and an N-acetyltransferase (*orf29*) indicates that the Ac–Val–Ala (6) moiety is synthesized by cluster 3. The adenylation (A)-domain specificity prediction by antiSMASH and other prediction tools revealed that *orf32* is incorporating Val, while *orf23* revealed unclear results from including Ala, Val, Trp, methionine (Met) or lysine (Lys).^27^

*Orf22* carries two A-domains, one with a prediction for threonine (Thr), while the other revealed the same unclear specificity as the one in *orf23*. Alignment of the two undefined A-domains from *orf22* and *orf23* revealed a 90% identity (Fig. S12), suggesting they have similar function. To reveal their role in the malevonin biosynthesis, *orf22* it was deleted from cluster 3. As a result, production of malevonin in the deletion mutant *S. albus* Del14_cluster3_delOrf22 was completely abolished and no new compound was observed, indicating that *orf22* is essential in the biosynthesis of malevonin (Fig. 2(E)).

The performed experiments indicate that malevonin biosynthesis is equally dependent on the Trp-derived C7 precursor from mansouramycin biosynthesis and the introduced cluster 3. A biosynthetic pathway was proposed starting with the formation of the acetylated dipeptide by the NRPS domains and the *N*-acetyltransferase in cluster 3, followed by the attachment of the substituted fluorine, which is derived from two moieties of the C7 precursor (5) from mansouramycin biosynthesis (Fig. 3). The assembly of the substituted fluorene scaffold and attachment to the dipeptide is yet unknown. A possible mechanism involves P450-mediated crosslinking, similar to that observed in other natural products such as arylomycins and cittilins.^21,28,29^ Orf31 in cluster 3 is annotated as a P450 enzyme and may catalyse an oxidative step during the formation of the fluorene core. Whether this process is enzymatically controlled or occurs spontaneously, however, remains unclear and will be explored in future studies. Biological activity testing of malevonin against bacterial and fungal strain, as well as cytotoxicity test were negative.

### Heterologous production of mansevorone

**Fig. 4:**
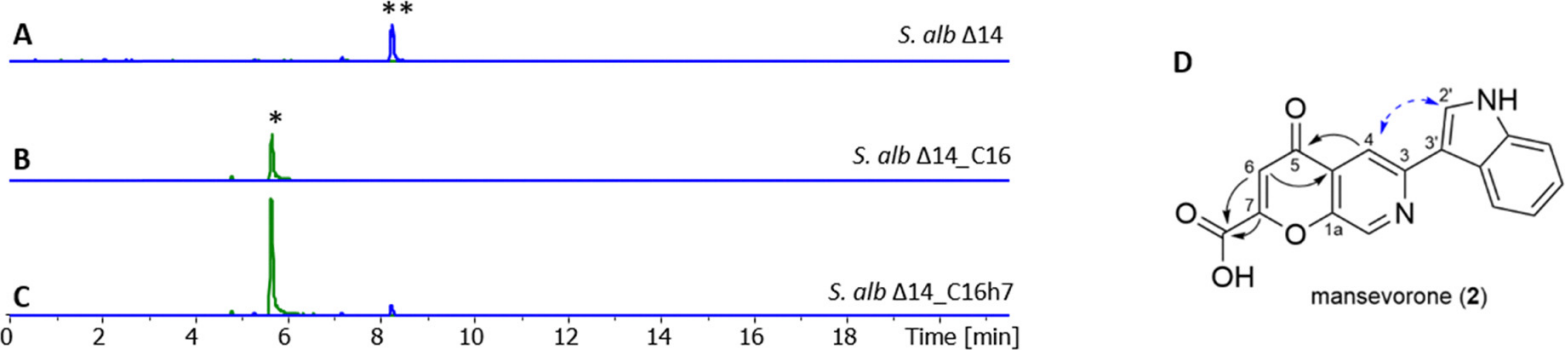
Extracted ion chromatograms showing mansevorone (2) and mansouramycin D (*) production in *S. albus* Del14 (A), *S. albus* C16 harboring cluster 16 (B) and in *S. albus* C16h7 harboring cluster16 and pRT801_ampery_AfsR16 for SARP overexpression (C). The mansevorone structure and key HMBC correlations (
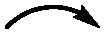
) and NOESY correlation (
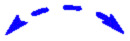
) are shown in D.

By working on the expression of a terpenoid BGC, designated as cluster 16, from a cosmid library constructed for the uncharacterized *Streptomyces* strain LV45-129 (GenBank acc. nr. CP023695.1, CP975_33055 – CP975_33225),^30^ we discovered another compound containing elements from mansouramycin D. Expression of the cluster 16 in *S. albus* Del14 led to an orange coloration of the culture and the production of a new compound whose proton adduct [M + H]+ had *m*/*z* = 307.072 (Fig. S13 and Fig. 4(A), (B)).

The production titres were insufficient for isolating the compound, requiring a refactoring of the cluster expression. Revisiting cluster 16, we identified a 2844-nucleotide gene encoding a 948-amino acid protein AfsR16 (CP975_33130) that was classified as a member of the SARP (*Streptomyces* Antibiotic Regulatory Protein) family through a BLAST search (Table S7). SARP regulators are transcriptional activators that directly influence the transcription of secondary metabolite biosynthesis genes; hence we aimed to overexpression of the gene to increase production using the pRT801 plasmid, which contains a strong synthetic promotor TS81.^31,32^ The resulting expression vector pRT801_ampery_AfsR16 was introduced into *S. albus* Del14 harboring cluster 16 *via* bacterial conjugation, generating the recombinant strain *S. albus* Del14_16h7. Subsequent determination of the production titre of the target compound associated to the [M + H]+ ion at *m*/*z* = 307.0712 resulted in a fivefold increase in target compound, indicating that the SARP regulator encoded by pRT801_ampery_AfsR16 plays a critical role in promoting the biosynthetic pathway of mansevorone in *S. albus* Del14_C16 (Fig. 4(C)).

The increase of production titre enabled the successful isolation of mansevorone after large-scale cultivation of *S. albus* Del14_C16h7 under the described conditions yielding 1 mg of the target compound, which was sufficient for subsequent NMR analysis (Table S8 and Fig. S14–S22).

The molecular formula was calculated as C_17_H_10_N_2_O_4_ based on [M + H]^+^ = 307.0712 Da Δ − 0.3 ppm (calc. 307.0713). The ^1^H-Proton NMR and edited-HSQC revealed eight methine signals in the downfield indicating a highly conjugated structure. In combination with COSY, ^13^C-HMBC and ^15^N-HMBC, 7 of these signals were assigned to an indole and a pyridine moiety. NOESY correlation between 2′-CH and 4-CH and HMBC correlations from 2′-CH to 3-C indicated that both moieties are connected between 3′-C and 3-C, revealing a structure similar to mansouramycin D without the MAB moiety (Fig. 4(D)). The new structural feature was calculated as C_4_H_2_O_4_ and assigned to 2-hydroxy-4-oxo-2-butenoic acid using HMBC correlations. The calculated number of 14 ring double bond equivalents and the chemical shift of 1a-C at *δ*_C_ = 150.5 ppm indicate a cyclic structure forming a 4-pyrone-2-carboxylic acid unit. Comparison of the calculated shift with the experimental data confirmed cyclization *via* C7-OH rather than cyclization *via* C7a-COOH. The structure was confirmed through MS/MS fragmentation, with experimental results compared to predicted outcomes generated by CFM-ID 4.0. (Fig. S24).^33–36^ The resulting structure is forming a rare 7-azachromone core and was named mansevorone (Fig. 4(D)).

### Mansevorone biosynthesis is encoded by *S. albus* Del14

Mansevorone shares structural similarities with mansouramycin D, but features a 4-pyrone-2-carboxylic acid unit in place of the MAB moiety. This suggests that mansevorone (2) may result from an interaction between cluster 16 and the native mansouramycin BGC from *S. albus* Del14. To determine whether the mansouramycin BGC is involved in mansevorone synthesis, cluster 16 was transferred into the mansouramycin BGC-deficient strain *S. albus* Del15. No production of mansevorone or any other new compound was observed in *S. albus* Del15_C16, indicating that mansevorone production is dependent on the presence of the mansouramycin BGC.

**Fig. 5:**
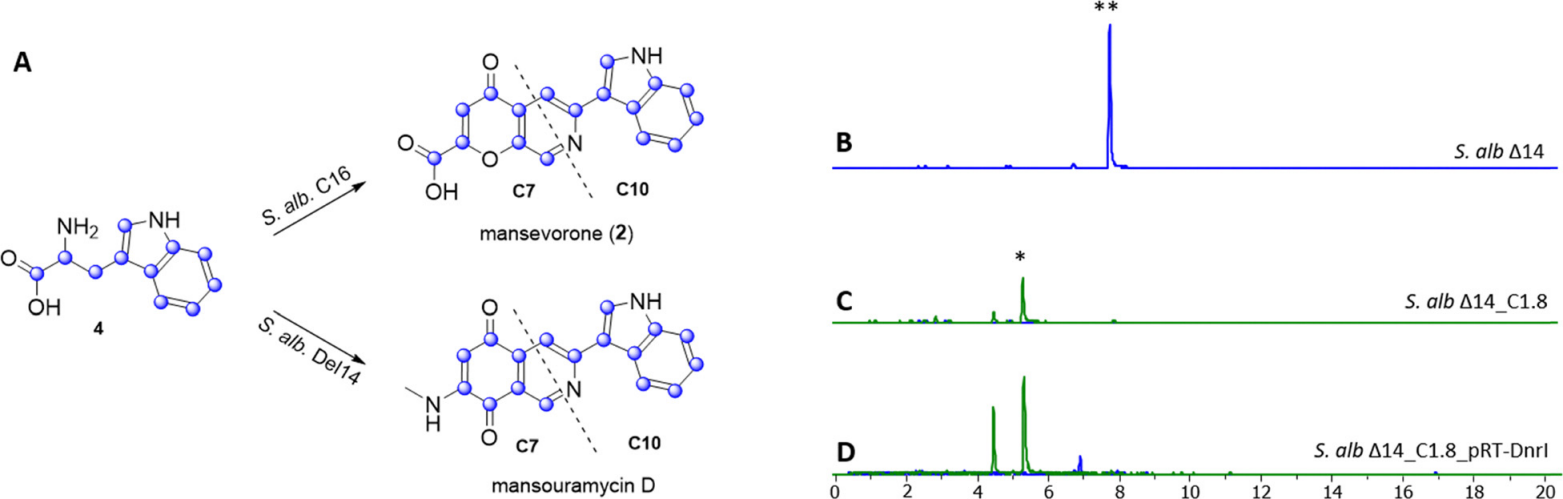
^13^C_11_-labeled tryptophan (blue dots) is incorporated by *S. albus* Del14_C16 into mansevorone and by *S. albus* Del14 into mansouramycin D; both compounds contain the similar C10 fragment but differ in the C7 fragment, while all fragments are synthesized from tryptophan (A). Extracted ion chromatograms showing mansevorone (*) and mansouramycin D (**) production in *S. albus* Del14 (B), *S. albus* Del14_C1.8 harboring cluster C1.8 (C) and in *S. albus* Del14_C1.8_rRT-DnrI harbouring cluster16 and pRT801_ampery_DnrI for SARP overexpression (D).

To further investigate the role of Trp in mansevorone biosynthesis, *S. albus* Del14_C16h7 was fed with ^13^C_11_-labeled tryptophan over the course of seven days. HPLC-MS analysis of the extracts revealed, in addition to the mansevorone ion at (*m*/*z* = 307.0712), additional ions with *m*/*z* values ranging from 313 to 324 Da, corresponding to mass shifts of +6 to +17 Da (Fig. S23). The most intense mass peaks were observed at *m*/*z* = 317.1049 and 324.1283 Da, indicating mass shifts of +10 and +17 Da, respectively, suggesting the involvement of two main biosynthetic precursor fragments, C7 and C10, both derived from Trp. The structural similarities between mansevorone and mansouramycin D suggest that the C10 fragment corresponds to the indole moiety, along with C-3 and C-4, which is likely attached in a similar manner to that proposed in the final biosynthetic steps of mansouramycin D (Fig. 5(A)). The 4-pyrone-2-carboxylic acid moiety, along with C-1, is derived from a different C7 precursor than the one used in mansouramycin D biosynthesis, suggesting a novel biosynthetic pathway.

The analysis of cluster 16 to identify potential genes involved in the biosynthesis of precursor fragments was unsuccessful. A possible explanation is that the SARP regulator AfsR from cluster 16 may function as a global regulator, influencing native biosynthetic genes in *S. albus* Del14 which activated mansevorone biosynthesis. Supporting evidence was found when we revisited previous heterologous expression studies of various gene clusters containing SARP regulators to examine whether they also produced mansevorone. Among these studies, cluster C1.8, which encodes a PKS-I compound from a cosmid library constructed for *Streptomyces kitasatoensis* (unpublished data), produced a compound with the same mass as mansevorone in *S. albus* Del14_C1.8_pRT-DnrI (Fig. 5(B)–(D)). The identity of mansevorone was confirmed through matching retention time, UV/VIS spectra, and MS/MS fragmentation patterns (Fig. S24 and S25).

Similar to the previous case, overexpression of the SARP regulator DnrI on cluster C1.8 led to increased production of mansevorone (Fig. 5(D)). Cluster 16 and cluster 1.8 and their SARP regulators originate from two unrelated strains and share very low homology (Fig. S26–S28). However, AsfR and DnrI are both from the same class of AfsR/DnrI/RedD of global transcriptional regulators and seem to activate mansevorone biosynthesis in *S. albus* Del14. Mansevorone production in *S. albus* Del14 only containing the SARP regulator containing plasmid pRT801_cat_ampery_Sk1.8_DnrI or pRT801_ampery_AfsR16 without the respective clusters C1.8 and 16 could not be observed.

This suggests that there is a more complex regulatory network involving regulatory genes on cluster C1.8 and 16 that are influenced by the SARP gene. However, the production of mansevorone after the heterologous transfer of two different gene clusters containing distinct SARP regulators strongly indicates that the biosynthetic genes for mansevorone are located in the genome of their heterologous host *S. albus* Del14.

Since biosynthesis of mansevorone is likely encoded in the native host *S. albus* Del14 and only expressed in the right conditions, a biosynthetic proposal cannot be made at this stage. A close examination of the biosynthetic mechanism will be conducted in future studies.

### Heterologous production of 5′Cl-mansouramycin

**Fig. 6:**
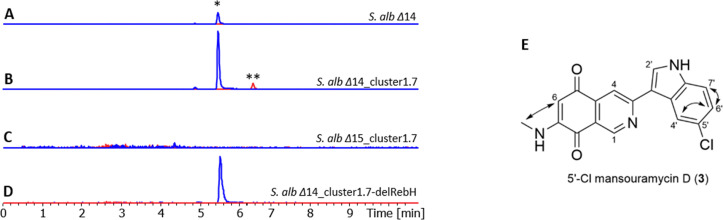
Production of mansouramycin D (*) and 5′-Cl-mansouramycin D (**) in *S. albus* Del14 chassis strain (A), the halogenase gene containing cluster 1.7 in *S. albus* Del14 (B), the mansouramycin cluster deficient strain *S. albus* Del15 (C) and in the RebH deletion mutant *S. albus* Del14_cluster1.7-delRebH (D). The structure of 5′-Cl-mansouramycin D and key COSY correlations (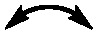) are shown in E.

During the investigation of additional heterologously expressed BGCs containing SARP regulators in *S. albus* Del 14, no further strains were found to produce mansevorone (5). However, heterologous expression of an NRPS cluster from *S. libani* (cluster 1.7, unpublished data) in *S. albus* Del14 led to the production of a novel compound with [M + H]^+^ ion at *m*/*z* = 338.0697. This compound displayed a characteristic chlorinated isotopic pattern and an MS/MS fragmentation profile similar to that of mansouramycin D, with a difference of 34 Da (Fig. 6(A), (B) and Fig. S32, S33).

The molecular formula was calculated as C_18_H_13_ClN_3_O_2_, based on the high-resolution mass 338.0697 [M + H]^+^, confirming the presence of single chlorine atom, likely attached to the mansouramycin D scaffold. To determine the site of chlorination, the compound was produced and isolated from *S. albus* Del14_cluster1.7 yielding 0.3 mg. Due to insufficient amounts of compound, HMBC and ^13^C NMR spectra could not be obtained. Nevertheless, ^1^H-NMR, COSY and edited-HSQC spectra (Fig. S29–S31) revealed that, all proton signals except one closely resembled those of mansouramycin D (Table S9). The missing proton signals was located in the phenyl ring of the indole moiety that is part of the mansouramycin D structure. Analysis of ^1^H-NMR revealed three key signals: a singlet (4′-C), a doubled (7′-C) and a doublet of doublets (6′-C), with COSY correlation confirming adjacency of 7′-C and 6′-C. These data pointed to possible chlorination at either the 5′ or 6′ positions of the indole ring. Comparison of the experimental and the empirically predicted chemical shifts by ACD/Spectrus Processor (version 2024) supported chlorination at the 5′-C position, leading to the assignment of the compound as 5′-Cl-mansouramycin (Fig. 6(E)).

A chlorinated version of mansouramycin D had not been previously observed in *S. albus* Del14, suggesting that the chlorination is likely mediated by genes encoded within the NRPS cluster1.7. This hypothesis was supported by the absence of both 5′-chloromansouramycin and mansouramycin D production when cluster 1.7 was expressed in the mansouramycin BGC deficient strain *S. albus* Del15 (Fig. 6(C)).

A potential gene responsible for chlorination, encoding a tryptophan halogenase enzyme RebH (Fig. S34), was identified through BLAST analysis of the cluster 1.7. Deletion of *rebH* and heterologous expression of the resulting construct, *S. albus* Del14_cluster1.7-delRebH, resulted in the depletion of 5′-chloromansouramycin production, with only mansouramycin D detected (Fig. 6(D)). This indicates that RebH is involved in the indole chlorination of mansouramycin D. Since RebH is a tryptophan halogenase and the final biosynthetic steps in the mansouramycin biosynthesis are known to exhibit some flexibility, we propose that chlorination occurs on the free tryptophan prior to its conversion to the C10 precursor which is used in the late-stage biosynthesis of 5′-chloromansouramycin.

This finding highlights the biosynthetic flexibility of the mansouramycin pathway, particularly during its final steps. Such flexibility offers an opportunity for the rational design of novel mansouramycin derivatives through structural modifications at the C3 and C4 positions, potentially enhancing their antitumor properties.

### Antimicrobial susceptibility test

Mansevorone and malevonin were tested against a panel of microorganisms and cells for cytotoxicity, however no activity could be observed. 5′-Cl-mansouramycin was not tested due to insufficient amounts.

## Conclusions

Novel compounds with structure elements from mansouramycins were discovered after heterologous expression of three distinct BGCs, indicating a biosynthetic interaction between the native mansouramycin genes of *S. albus* Del14 and the introduced genes. This resulted in the rare fluorene and azachromone scaffolds of malevonin (1) and mansevorone (2) and a 5′-chlorinated version of mansouramcin D (3). The dynamic interaction of the mansouramycin biosynthesis with heterologously expressed clusters is intriguing and raises many questing regarding the final biosynthetic steps of mansouramycin D, the interaction of mansouramycin precursors and the regulation of gene expression. The mansouramycin pathway in *S. albus* Del14 is very responsive to heterologously introduced BGCs, which might influence the expression of compounds. This has to be taken in consideration when using *S. albus* Del14 as heterologous expression host. The interaction of biosynthetic gene cluster is the foundation of the cluster evolution and the huge variety of natural products from *Streptomyces*. Understanding the biosynthetic mechanisms of mansouramycin biosynthetic crosstalk could enable new strategies for targeted production of novel compounds.

## Experimental

### General experimental procedures

All strains, plasmids, BACs and cosmids utilized in this study are listed in Table S1. *Escherichia coli* strains were cultured in Luria Bertani (LB) medium.^37^ Soya flour mannitol agar (MS agar) was used for the cultivation of *Streptomyces* strains, supporting both sporulation and conjugation. Tryptic soy broth (TSB; Sigma-Aldrich, St. Louis, MO, USA)^38^ and liquid DNPM medium 40 g L^−1^ dextrin, 7.5 g L^−1^ soytone, 5 g L^−1^ baking yeast, and 21 g L^−1^ MOPS, (pH 6.8) was utilized for expression and secondary metabolite production. Antibiotics such as kanamycin, apramycin, hygromycin, and nalidixic acid were added when necessary.

### Genome mining and bioinformatic analysis

Genomes were screened for secondary metabolite biosynthetic gene clusters using the antiSMASH online tool.^27^ Genetic data was analyzed with the Geneious prime 2022.2.2 software.^39^ Selected genes were further analyzed using the BLAST online tool from the National Center of Biotechnology Information (https://www.ncbi.nlm.nih.gov/BLAST/) and the Universal Protein Resource (UniProt) (https://www.uniprot.org/blast).

### Isolation and manipulation of DNA

BAC and cosmid isolation from the constructed genomic library of *Streptomyces* LV45-129, *S. kitasatoensis* and *S. libani*, DNA manipulation, transformation into *E. coli* and intergeneric conjugation between *E. coli* and *Streptomyces* were performed according to standard protocols.^37,38,40^ Purification of cosmids was carried out using the BACMAX™ DNA purification kit (Lucigen, Middleton, WI, USA). All restriction endonucleases were used according to manufacturer's recommendations (New England Biolabs, Ipswich, MA, USA). All of the Primers used in this study are listed in Tables S3 and S4.

### Construction of the BAC vector containing cluster 3

Two cosmids 01A06 and 12F02 from the *S. kitasatoensis* library, containing left and right parts of the cluster 3, were used in the homologous recombination experiment,^7,41^ using yeast^42^ (Table S1) and pCLY10 vector^3^ (Table S2) containing left and right homologous arms to the both cosmids, integrase gene and origin of replication for *Streptomyces* strains, in order to obtain full cluster 3 as one piece. Correct transformants were verified by the restriction analysis and sequencing of the isolated BAC DNA (Table S2). As a result, the pBACcluster-3 expression vector was obtained.

### The deletion of the *orf22* gene, coding for NRPS-1, in cluster 3

#### Modification of the BAC containing cluster 3 by the Red/ET recombination

Red/ET recombination technology was used in order to delete the orf22 gene. For this purpose, linear DNA fragments flanked by suitable homology arms were generated by PCR using Cluster3ReddelNRPS_F and Cluster3ReddelNRPS_R primers (Table S3) and fragment containing hygromycin resistance gene. The PCR reactions were carried out with Phusion DNA polymerase (Thermo Fisher Scientific) according to the manufacturer's protocol. PCR products were concentrated by ethanol precipitation prior to further use. In general, 300 µl overnight culture of *E. coli* GB05-red (Table S1) cells harboring the cluster 3 BAC vector (Table S2) to be modified were inoculated into 15 ml of LB, and cultivated on a shaker at 37 °C and 200 rpm for 2 hours. Thereafter, 400 µl of 10% l-rhamnose was added to the culture to induce the expression of the recombinases. Cultivation was then continued for 45 min. The cells were subsequently harvested by centrifugation, washed twice with ice-cold distilled H2O, and resuspended in 600 µl of 10% ice-cold glycerol. The PCR product was mixed with the electrocompetent cells, which were then transferred to an ice-cold electroporation cuvette (1 mm). The mixture was subsequently electroporated at 1800 V (Eppendorf electroporator), followed by the addition of 750 µl LB. The cells were cultivated at 37 °C for 90 min before the culture was transferred to LB agar plates with hygromycin. The plates were incubated at 37 °C overnight. Correct transformants were verified by the restriction analysis and sequencing of the isolated BAC DNA using the Cluster3checkF and Cluster3checkR primers (Table S3). As a result, the pBACcluster-3_Delorf22 vector was obtained.

#### Construction of the *S. albus* Del14_cluster3_delOrf22 mutant

The pBACcluster-3_Delorf22 (Table S2) gene disruption vector was transferred from *E. coli* ET12567/pUB307 cells into *S. albus* Del14 cells by means of conjugation. Transconjugants were selected for resistance to hygromycin (50 µg ml^−1^) and apramycin (50 µg ml^−1^). As a result, the *S. albus* Del14_cluster3_delOrf22 was obtained (Table S1).

### Construction of the SARP expression vector

The SARP expression vector pRT801_ampery_AfsR16 and pRT801_ampery_DnrI were constructed based on the phage BT1 integrative vector pRT801, incoporating the SARP gene under the control of the strong, constitutive promoter TS_81. Amplification of the SARP gene was performed using the primers shown in Table S4, which included additional NdeI and EcoRV restriction sites to facilitate subsequent cloning. AsfR16_Terp_For and C1.8_DnrI_act-ML-f also contained a sequence a 22bp sequence derived from the upstream region of the SARP gene, encompassing the ribosomal binding site. Successful integration of the SARP gene into the vector was confirmed by sequencing using the sequencing primers shown in Table S4.

### Construction of the BAC1.7-delRebH-delAp vector

The halogenase *rebH* gene was functionally inactivated by an in-frame deletion of its main coding region. First, the ampicillin resistance gene was amplified using primers delRebH-f and delRebH-r (Table S3). The resulting PCR product was introduced into the BAC1.7 vector *via* Red/ET-mediated homologous recombination to replace the central portion of the *rebH* gene, yielding the intermediate construct BAC1.7-delRebH-Ap. The ampicillin resistance cassette was subsequently removed by *MssI* restriction digestion followed by self-ligation. The final plasmid, designated BAC1.7-delRebH-delAp, was introduced into *S. albus* Del14 strain *via* conjugation to assess the effect of the *rebH* gene deletion.

### Metabolic extraction and analysis

All *Streptomyces* strains were precultured in 25 mL of TSB medium for 24 h before 1 mL was taken from this seed culture to inoculate 100 mL of the DNPM production medium. These cultures were incubated for seven days at 28 °C. Metabolites were extracted with equal amounts of butanol or ethyl acetate from the supernatant of the culture broth, evaporated and dissolved in methanol. HPLC-MS analysis was performed by separating 1 µL of the extract using a Dionex Ultimate 3000 UPLC (Thermo Fisher Scientific, Waltham, MA, USA), a 10-cm ACQUITY UPLC BEH C18 column, 1.7 µm (Waters, Milford, MA, USA) and a linear gradient of 0.1% formic acid solution in acetonitrile against 0.1% formic acid solution in water from 5% to 95% in 18 min at a flow rate of 0.6 mL min^−1^. Samples were analyzed using an amaZon speed mass spectrometer or maXis high resolution LC-QTOF system (Bruker, USA). Data were collected and analyzed with the Bruker Compass Data Analysis software, version 4.1 (Bruker, Billerica, MA, USA). Monoisotopic mass was searched in the natural products database DNP (Dictionary of Natural Products).^43^

### Malevonin, mansevorone and 5′-Cl-mansouramycin isolation

*Streptomyces albus* Del14 cluster3, *Streptomyces albus* C16h7 and *Streptomyces albus* Del14_cluster1.7 each were precultured in 6 flasks, each containing 25 mL of TSB, for 24 h. Subsequently, 100 flasks, each containing 100 mL of DNPM (*S. albus* Del14_C16h7 and Δ14_cluster1.7) or SG production medium (*S. albus* Del14_cluster3), were inoculated with 1 mL of the seed culture. The cultures were incubated for 7 days at 28 °C. The mycelial portion was separated by centrifugation, and metabolites were extracted from the supernatant with equal amount of butanol.

#### Malevonin purification

Malevonin precipitated from the crude extract when dissolved in methanol. The precipitant was collected, washed with hexane and dried, resuling in the pure compound.

#### Mansevorone purification

In a first step, mansevorone was isolated from the crude mixture by flash chromatography using a Biotage Isolera system (Biotage, Uppsala, Sweden) equipped with CHROMABOND Flash RS 330 C18 ec cartrige (MACHEREY-NAGEL, Düren, Germany) and gradient elution using a mixture of water and methanol as solvents. The enriched fractions were pooled and subjected to size exclusion cloumn chromatography using Sephadex LH-20 (GE Healthcare, Chicago, USA) resin and methanol as eluent. Semi-preparative reversed phase HPLC purification was perfomred on a Agilent 1260 infinity (Agilent Technologies, Kalifornien, USA) equipped with a Synergi C18 column (Phenomenex, Aschaffenburg, Germany) and gradient elution using a 0.1% formic acid solution in acetonitrile and water as the mobile phase. Fractions containing mansevorone were detected by LC–MS analysis as described above, pooled together and evaporated for further experiments.

#### 5′-Cl-mansouramycin purification

5′-Cl-mansouramycin was purified by size exclusion chromatography using a Sephadex LH-20 column (GE Healthcare, Chicago, USA) and methanol as a solvent. Preparative HPLC of the enriched fractions was carried out on a Waters Autopurification System equipped with a Nucleodur C18 Htec 250/21-C18 5 µm column (Macherey-Nagel, Düren, Germany) and gradient elution using a 0.1% formic acid solution in acetonitrile and water as the mobile phase. Fractions containing 5′-Cl-mansouramycin were detected by LC–MS analysis as described above, pooled together and evaporated for further experiment.

### Feeding studies

l-Tryptophan ^13^C_11_ was purchased from Euroisotope. For feeding experiments, l-Tryptophan was prepared as a stock solution at a concentration of 5 mg mL^−1^. From this stock solution, 125 µL was added to 25 mL cultures at inoculation and again after 24, 48, and 72 hours of cultivation. After 96 h, the culture supernatant was extracted with an equal volume of butanol. The solvent was evaporated under a nitrogen stream, and the residue was dissolved in 1 mL of methanol. The resulting extract was subsequently analyzed using LC–MS analysis as described above.

### Antimicrobial susceptibility test

Minimum inhibitory concentrations (MICs) were determined according to standard procedures. Single colonies of the tested strains were suspended in cation adjusted Müller–Hinton broth to achieve a final inoculum of approximately 104 CFU ml^−1^. Serial dilutions of mansevorone (0.03 to 64 µg mL^−1^) were prepared in sterile 96-well plates before the strain suspension was added. Growth inhibition was assessed after overnight incubation (16–18 h) at 30–37 °C. A panel consisting of the following strains was tested: *B. subtilis* DSM-10, *S. aureus* Newman, *Mycobacterium smegmatis* MC2155, *Citrobacter freundii* DSM-30039, *E. coli* BW25113 (wt), *E. coli* JW0451-2 (ΔacrB), *Pseudomonas aeruginosa* PA14 DSM-19882, Acinetobacter baumanii DSM-30008, *Mucor hiemalis* DSM-2656, *Pichia anomala DSM-6766*, *Cryptococcus neoformans* DSM-11959, *Candida albicans* DSM-1665, CHO-K1 and HepG2.

### Nuclear magnetic resonance spectroscopy (NMR)

The chemical structures of all the compounds were determined *via* multidimensional NMR analysis. ^1^H-NMR, ^13^C-NMR, and 2D spectra were recorded at 500 MHz (^1^H)/126 MHz (^13^C), conducted in the Bruker Avance Neo 500 MHz, equipped with a Prodigy Cryo-probe. Samples were dissolved in methanol-d_4_ or dimethyl sulfoxide-d_6_. Chemical shifts are reported in ppm relative to tetramethylsilane; the solvent was used as the internal standard. Coupling constants are reported in Hertz (Hz). Multiplicity is reported with the usual abbreviations (s: singlet, br s: broad singlet, d: doublet, dd: doublet of doublets, ddd: doublet of doublet of doublets, t: triplet, dt: doublet of triplets, q: quartet, p: pentet, dp: doublet of pentets, m: multiplet).

## Author contributions

A. L., M. S., L. H., P. O. and M. L. designed experiments. M. S., L. H., P. O., M. L., A. P., P. C. and C. R. performed experiments. M. S. solved NMR structures, M. S. and P. O. wrote the manuscript. All authors edited the manuscript. A. L. and J. Z. supervised the work.

## Conflicts of interest

There are no conflicts to declare.

## Supplementary Material

CB-007-D5CB00235D-s001

## Data Availability

All experimental data supporting the findings of this study are provided in the supplementary information (SI). Supplementary information: detailed descriptions of the strains and plasmids used, gene cluster annotations, primer sequences, and biosynthetic pathway analyses. Raw and processed NMR spectra, LC–MS data, MS/MS fragmentation profiles, UV-Vis spectra, and results of ^13^C-labelled precursor feeding experiments for the compounds malevonin, mansevorone, and 5′-Cl-mansouramycin D. The DNA sequences of key biosynthetic genes (*e.g.*, *rebH*, *afsR16*, *dnrI*) used in gene deletions and overexpression experiments are also provided in the SI (Fig. S26–S28 and S34). Full lists of all strains and plasmids (Tables S1–S2), gene cluster compositions (Tables S5 and S7), and all NMR assignment Tables (S6, S8 and S9). See DOI: https://doi.org/10.1039/d5cb00235d.
