# Supplementary material for: Heterologous biosynthetic crosstalk with the native mansouramycin cluster in Streptomyces albus Del14 reveals unexpected metabolites
Source: RSC Chem Biol. 2025 Dec 3;7(2):240–9. doi: 10.1039/d5cb00235d (PMC12690601; doi:10.1039/d5cb00235d)
Supplement: CB-007-D5CB00235D-s001 [file CB-007-D5CB00235D-s001.pdf]

Supplementary Information (SI) for RSC Chemical Biology.
This journal is © The Royal Society of Chemistry 2025

Supplementary: Heterologous Biosynthetic Crosstalk with the Native Mansouramycin Cluster in Streptomyces albus Del14 Reveals Unexpected Metabolites

Marc Stierhof, Liliya Horbal, Patrick Oberhäuser, Anja Paluszak, Maria Lopatniuk, Peyton Cox, Christopher Ruf, Josef Zapp, Andriy Luzhetskyy

Table S1: Bacterial strains used in this study.

Strain	Description	Source
S. lividans Del8	Cluster free derivative of *S. lividans* TK24	Yousra A., et al.[1]
S. albus Del14	Cluster free derivative of *S. albus* J1074	Myronovskyi, M., et al.[2]
S. albus Del15	Derivative of *S. albus* Del14 with the deletion of mansouramycin biosynthetic pathway	Shuai, H., et al.[3]
Escherichia coli ET12567 pUB307	Donor strain for intergeneric conjugation	Flett, F. et al.[4]
Escherichia coli DH10β	General cloning strain	Grant, S.G., et al.[5]
Escherichia coli GB05-red	*E. coli* strain used for the Red/ET recombination	Zhang et al.[6]
Saccharomyces cerevisiae BY4742	Auxotrophic strain used for homologous recombination in yeast	Baker Brachmann, C., et al.[7]
S. albus Del14_cluster3	Derivative of *S. albus* Del14 harboring the cluster 3	this study
S. albus Del15_cluster3	Derivative of S. albus Del15 harboring cluster 3	this study
S. albus Del14_cluster3_delOrf22	Derivative of S. albus Del14 harboring the cluster 3 with deletion of orf22 gene	this study
S. albus Del15_C16	Derivative of *S. albus* Del15 harboring cluster 16	this study
S. albus Del14_C16	Derivative of *S. albus* Del14 harboring cluster 16	this study
S. albus Del14_C16h7	Derivative of S. albus Del14 harboring the cluster 16 and pRT801_ampery-AfsR16 plasmid	this study
Streptomyces LV45-129	The wild-type strain; the source of cluster 16	this study
S. albus Del14_C1.8	Derivative of *S. albus* Del14 harboring BAC_C1.8	this study
S. albus Del14_C1.8_pRT-DnrI	Derivative of *S. albus* Del14 harboring BAC_C1.8 and pRT801_cat_ampery_Sk1.8_DnrI	this study
S. albus Del14_cluster1.7	Derivative of *S. albus* Del14 harboring BAC1.7	this study
S. albus Del14_cluster1.7-delRebH	Derivative of *S. albus* Del14 harboring BAC1.7 with RebH deletion	this study
S. albus Del15_cluster1.7	Derivative of *S. albus* Del15 harboring BAC1.7	this study

Table S2: Plasmids and BACs used in this study**.**

Name	Despriction	Source
cluster 3	cluster originating from a COSMID library created for *Streptomyces kitasatoensis*	this study
cluster 3_ Delorf22	cluster 3 with deletion of NRPS gene orf22	this study
pCLY10	Shuttle vector for *E.coli*-yeast-actinomycetes	Bilyk O., et al.[8]
cluster 16	cluster originating from a COSMID library created for *Streptomyces strain LV45-129*	Oberhäuser P., et al.[9]
pRT801	Plasmid; BT1 integrative vector for Streptomyces strains; apramycin resistance	Gregory, M.A.[10]
pRT801_cat_ampery	Derivative of pRT801 containing ampicilin-erythromycin resistance cassette and strong synthetic promoter TS81	Shuai, H., et al.[3]
pRT801_ampery_AfsR16	Derivative of pRT801_cat_ampery, catalase gene was replaced with the SARP gene from cluster 16	this study
BAC_C1.8	Cluster originating from a COSMID library created for *Strepsomyces*	this study

	kitasatoensis	
pRT801_cat_ampery _Sk1.8_DnrI	Derivative of BT1 integrative vector pRT801_cat_ampery, catalase gene was replaced with the SARP gene from cluster 1.8	this study
BAC1.7	cluster 1.7	this study
BAC1.7-delRebH-delAp	cluster 1.7 with in-frame deletion of RebH halogenase gene	this study

Table S3: Primers used for deletion of genes.

Primer	Sequence	Description
Cluster3Reddel NRPS_F	TTCCAGATCCGCCGCGGGTGCTTCGGTGCCCCGGAGGCCGGCGAGGTCG TGGACGAAGGCGGCCAGCAGCTCAACGGGAATCCTGCTCTGCGAG	Forward primer for the deletion of the NRPS-1 gene in the cluster 3.
Cluster3Reddel NRPS_R	CTGATGACCGACGCGACGCCCCGGCAGACGACGATCGAGGAGAAGCGG CGCGCGCTGCTGGCCCTGCGGCTGTCTTGTAGGCTGGAGCTGCTTC	Reverse primer for the deletion of the NRPS-1 gene in the cluster 3.
Cluster3checkF	ATCAGACCGGTGCGCTCCAGG	Forward primer for checking the deletion of the NRPS-1 gene.
Cluster3checkR	ACCGACTGCTGAGTGAGGAAG	Reverse primer for checking the deletion of the NRPS-1 gene.
delRebH-f	CTCAAGGCGGCCTACGGTGACCGCATCAACGTCACTCTGGTGGAATCCG AGTTTAAACAGCTGTTCCGGGGATCCGTC	Amplification of ampicilin cassette for in frame deletion of RebH, forward.
delRebH-r	CGCCCGGAGCCGCGCGAGCTCGGCGCGCGCGTTCGTCGGGTCGATGTG CGGTTTAAACTGTAGGCTGGAGCTGCTTCG	Amplification of ampicilin cassette for in frame deletion of RebH, reverse.
RebH-chk-F	ATGGCGCGAAGAATCTGCCG	Sequencing of deletion construct for verification, forward.
RebH-chk-R	GAAGGGTGGCCATGGTGAGG	Sequencing of deletion construct for verification, reverse.

Table S4: Primers used for SARP overexpression.

Primer	Sequence	Description
AsfR16_Terp_For	CATATGGTCGAGCAACGGAGGTACGGACGTGGAGTTCC GGCTGCTCGG	AsfR amplification primer including rbs and NdeII restriction side
AsfR16_Terp_Rev	GATATCTCAGGTCCGCGCCATGAGCCGGACCAT	AsfR amplification primer including EcoRV restriction side
pRT_chk_For	TAGTTCCTTCGTCACCACAG	Primers for sequencing from pRT801 region
pRT_chk_Rev	TCACTCATTAGGCACCCCAG	Primers for sequencing from pRT801 region
AfsR16_chk1	CTCGACTGGTTCCACCGC	Primers for sequencing from AsfR Region
AfsR16_chk2	GTTTCAGCGACACCGGAGA	Primers for sequencing from AsfR Region
AfsR16_chk3	CGAACTGTCCGACGAGTCAC	Primers for sequencing from AsfR Region
AfsR16_chk4	CAGGACCTGGCGGATCATC	Primers for sequencing from AsfR Region
C1.8_DnrI_act-ML-f	AAAACATATGGTCGAGCAACGGAGGTACGGACATGCAC GCACTCCGGACG	DnrI amplification primer including rbs and NdeII restriction side
C1.8_DnrI_act-r	TTTTGATATCTCAGCACCCCCTCAGCAC	DnrI amplification primer including EcoRV restriction side
prt801_cat-exchange_chk_f	TGAAGGAGGAAGACGAAGCG	Primers for sequencing from pRT801 region

prt801_cat-exchange_chk_r	AGTCAGTGAGCGAGGAAGC	Primers for sequencing from pRT801 region
Sk1.8_DnrI_seq-1	TGGAGTCCTACGCCGAGG	Primers for sequencing from DnrI Region
Sk1.8_DnrI_seq-2	TTACTGATGGAGCTCGCC	Primers for sequencing from DnrI Region
Sk1.8_DnrI_seq-3	TACTTCGTCATGCAGCTCCT	Primers for sequencing from DnrI Region
Sk1.8_DnrI_seq-4	TGCACATCCAGCTGGGGC	Primers for sequencing from DnrI Region

Table S5: Proposed function of the genes from cluster 3 and similarity comparison to the *vzb* gene cluster.

cluster 3		***vzb cluster***	
**orf**	**proposed function**	**corresponding genes**	**% Similarity to cluster 3**
1	ATP-dependent protease HslVU (ClpYQ), peptidase subunit		
2	putative protein		
3	autoinducer-binding transcriptional regulator		
4	SARP		
5	SARP		
6	acyl-CoA_dehydrogenase	vzb5	49
7	Cyclohexane-1-carbonyl-CoA dehydrogenase	vzb6	66
8	Thioesterase	vzb14	58
9	sulfate adenyl transferase	vzb18	77
10	Sulfate adenylyltransferase subunit 2	vzb19	86
11	Adenylyl-sulfate kinase	vzb20	73
12	Sulfotransferase family protein	vzb21	65
13	Alpha-aminoadipate carrier protein LysW, vzb22	vzb22	67
14	acetyl-CoA_carboxylase_biotin_carboxylase, vzb23	vzb23	70
15	N-acetyl-gamma-glutamyl-phosphate_reductase, vzb24	vzb24	75
16	acetylglutamate kinase-like protein LysZ, vzb25	vzb25	72
17	acetylornithine_deacetylase, vzb26	vzb26	69
18	transketolase, vzb27	vzb27	62
19	1-deoxy-D-xylulose-5-phosphate_synthase	vzb28	70
20	Glyoxalase/bleomycin resistance protein/dioxygenase		
21	major_facilitator_transporter		
22	**NRPS-1**		
23	**NRPS-2**		
24	Argininosuccinate lyase/adenylosuccinate lyase	vzb2	57
25	PCP	vzb8	50
26	Aminotransferase, vzb9	vzb9	70
27	Butirosin biosynthesis protein H, N-terminal, Azi29	vzb10	69
28	Azi28	vzb11	69
29	**Acetyltransferase (GNAT) family protein (there is an acetyl group in the product)**	vzb12	49
30	Succinyl-diaminopimelate desuccinylase	vzb13	69
31	cytochrome_P450		
32	**NRPS-3**	vzb15	59
33	mbtH-like protein	vzb16	49
34	Putative protein		
35	LuxR family DNA-binding response regulator	vzb17	50
36	Putative protein	vzb0	14

37	Helix-turn-helix domain protein		
38	Hypothetical protein		
39	LysR family transcriptional regulator		
40	2-amino-3-ketobutyrate coenzyme A ligase		
41	Alcohol dehydrogenase		
42	AsnC family transcriptional regulator		
43	L-asparaginase		
44	Aspartate ammonia-lyase		
45	NAD-dependent malic enzyme		
46	TetR_family_transcriptional_regulator		
47	GCN5-related_N-acetyltransferase		
48	Putative membrane protein		
49	Protease HtpX		
50	Acyl-CoA dehydrogenase		

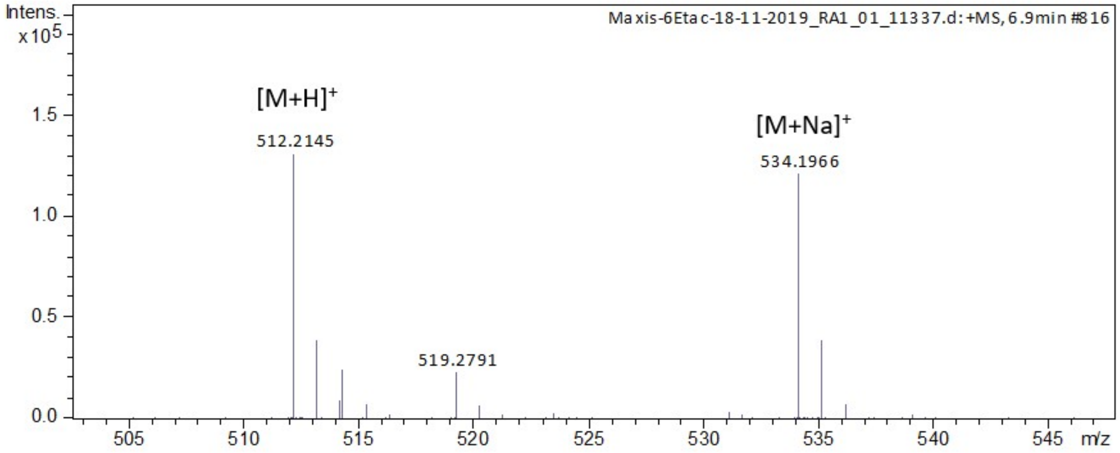

Figure S1: HRMS spectrum of malevonin.

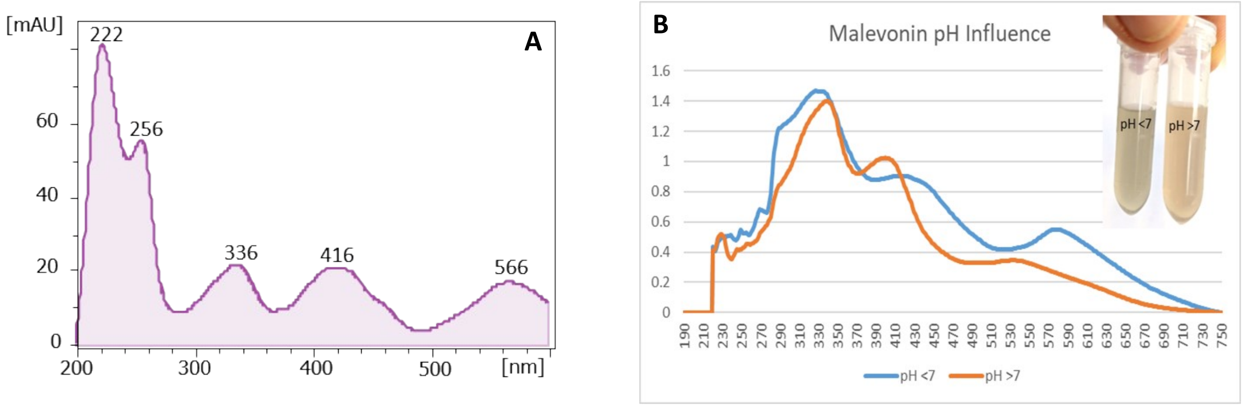

Figure S2: UV/VIS spectrum of malevonin extracted from the LC-MS run (**A**) and UV/VIS measurement of malevonin in DMSO/ water (50/50) at high and low pH (**B**).

Table S6: NMR data (500 MHz, DMSO-d6) of malevonin.

residue	no	δ(^{13}C) [ppm], type	δ(^{1}H) [ppm], mult (J)	COSY (H-)	HMBC (C-)
AHMFT*	1/8	172.3, 2xC			3,6
	1a/8a	115.1, 2xC			9-NH, 3/6
	2/7	157.7, 2xC			3/6, 2/7-NMe
	2/7-NH		8.94, 2	2/7-NMe	
	2/7-NMe	30.8 2xCH_3	2.93, s	2/7-NH	
	3/6	94.5, 2xCH	5.53. s	2/7-NMe	
	4/5	178.2, 2xC			3/6
	4a/5a	124.8, 2xC			3/6
	9	138.5, C			9-NH
	9-NH		9.89, s		
	OH		18.47, bs		
Ac	10	170.1, C			13-NH, 13, 11
	11	23.3, CH_3	1.87, s		-
Val	12	171.7, C			18-NH, 13.14.18
	13	58.2, CH	4.22. t (7.9)	13-NH,14	13-NH, 14, 15, 16
	13-NH		7.91, d (8.9)	13	
	14	31.3, CH	1.94, m (6.7)	13, 15, 16	13-NH, 13, 15, 16
	15	20.1, CH_3	0.84, d (6.7)	14	12, 13, 16
	16	19.0, CH_3	0.81, d (6.7)	14	12 ,13 ,15
Ala	17	170.2, C			9-NH, 18, 18-NH, 19
	18	49.6, CH	4.53, p (6.9)	18-NH, 19	18-NH, 19
	18-NH		8.16, d (7.0)	18	
	19	18.6, CH_3	1.38, d (7.0)	18	18-NH, 18

*9-amino-5-hydroxy-2,7-bis(methylamino)-1H-fluorene-1,4,8-trione (AHMFT)

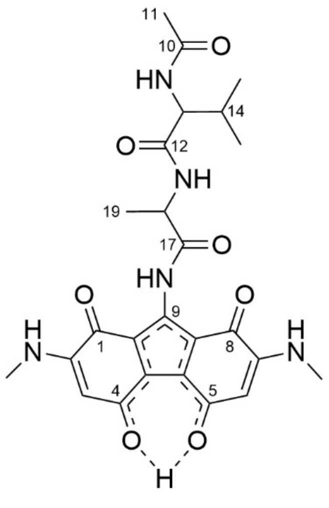

malevonin (**1**)

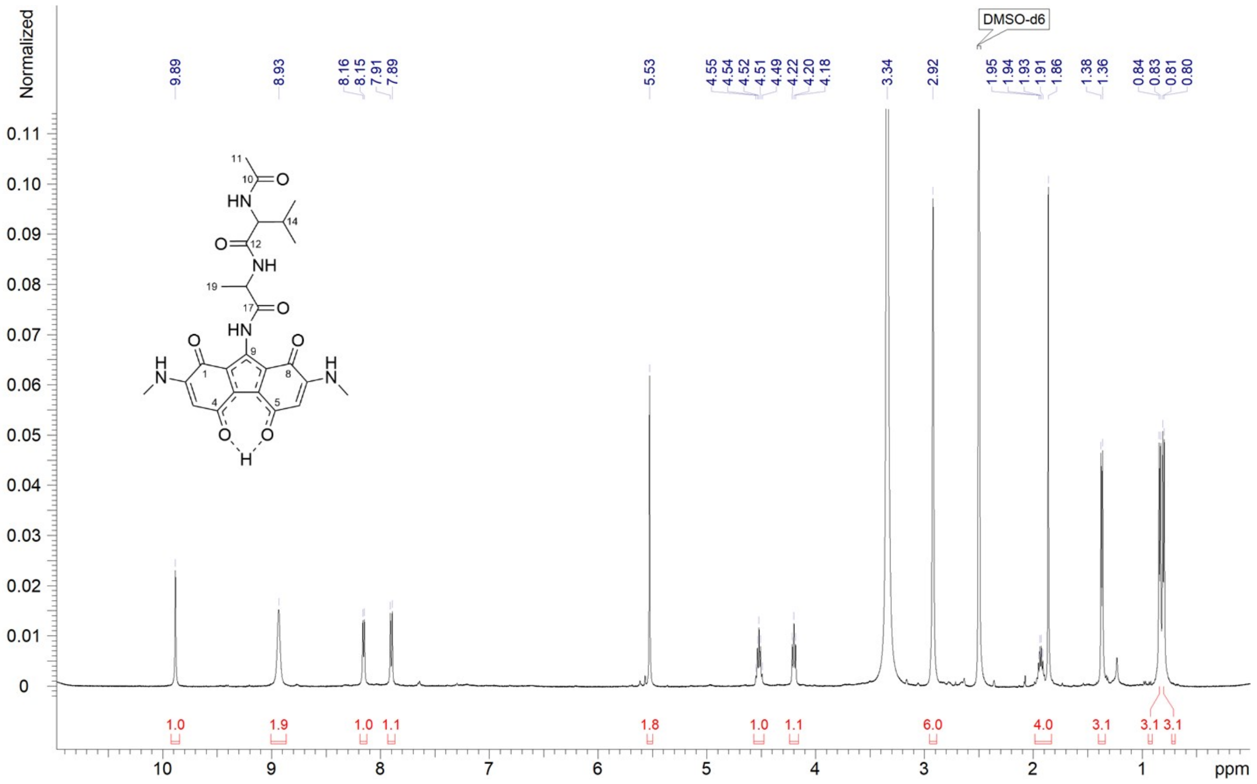

Figure S3. ^{1}H NMR spectrum (500 MHz, DMSO-*d6*) of malevonin.

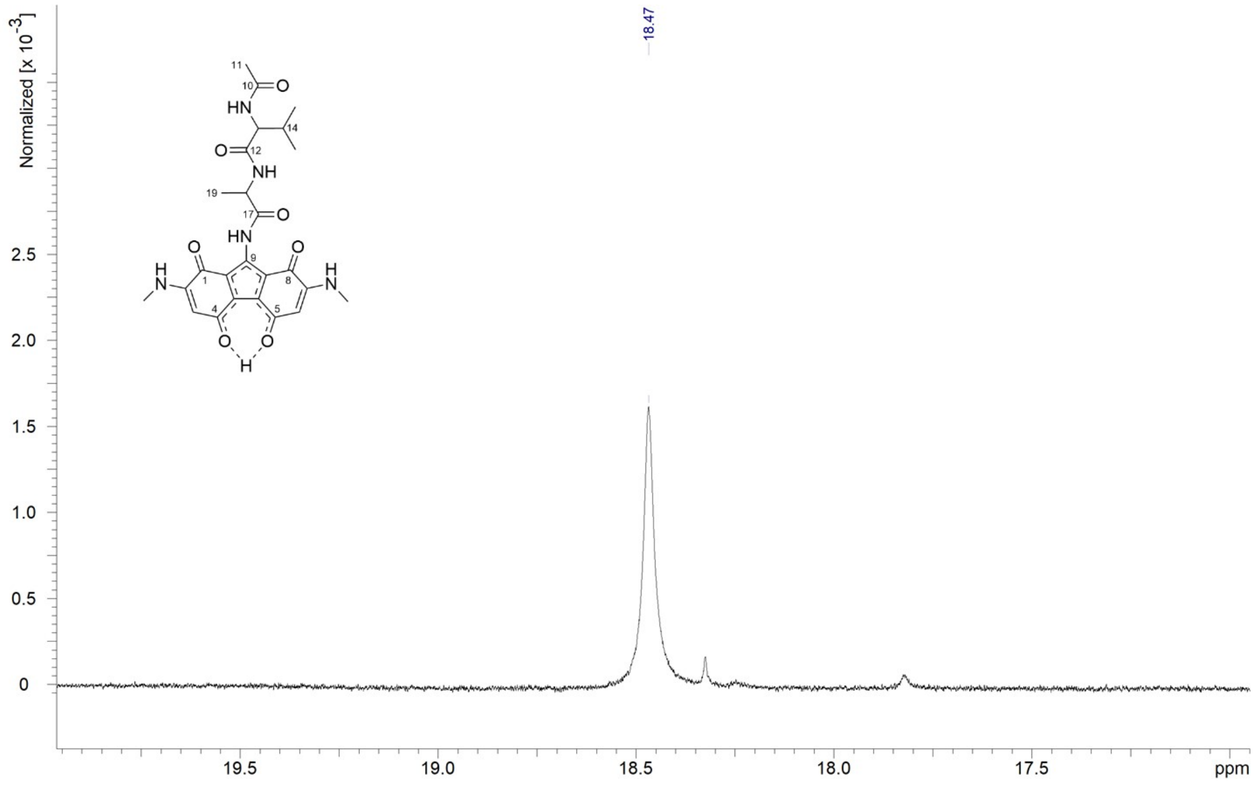

Figure S4: ^{1}H NMR spectrum (500 MHz, DMSO-*d6*) of malevonin showing the hydrogen bridge stabilized proton signal in the downfield.

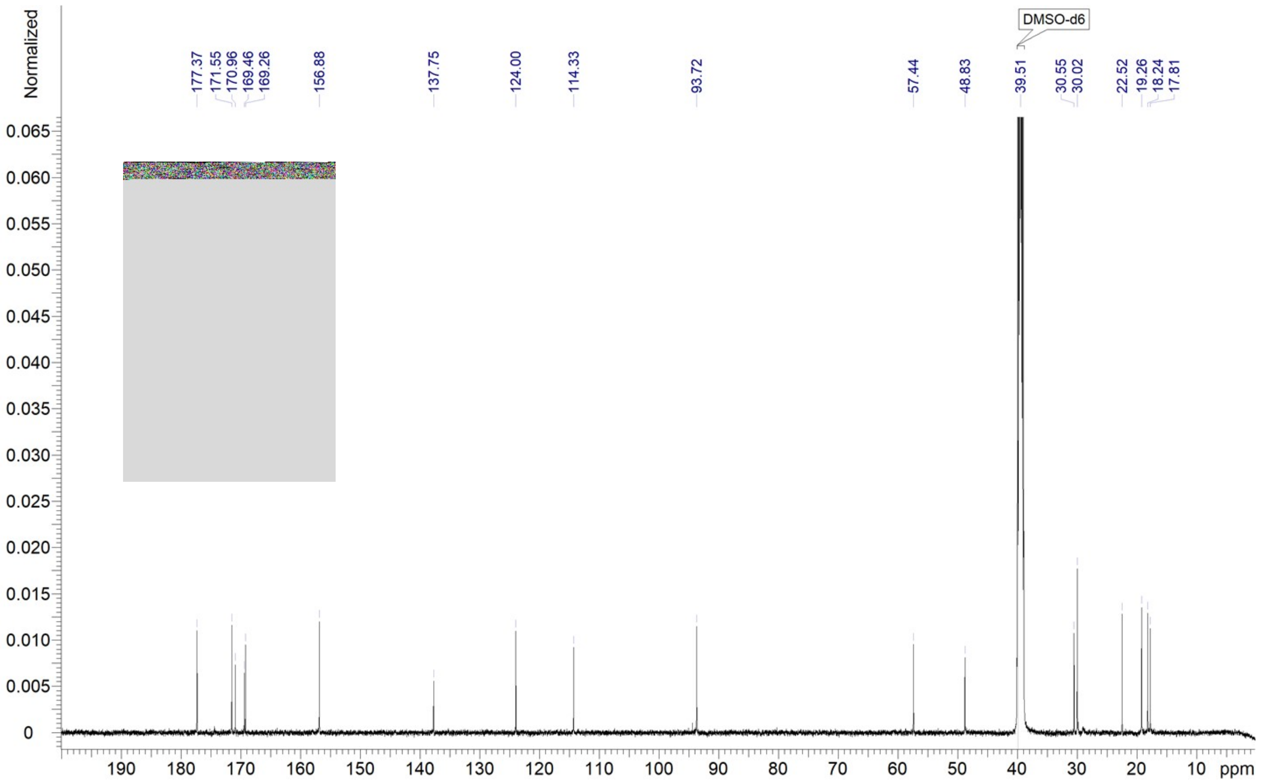

Figure S5: ^{13}H NMR spectrum (125 MHz, DMSO-*d*6) of malevonin.

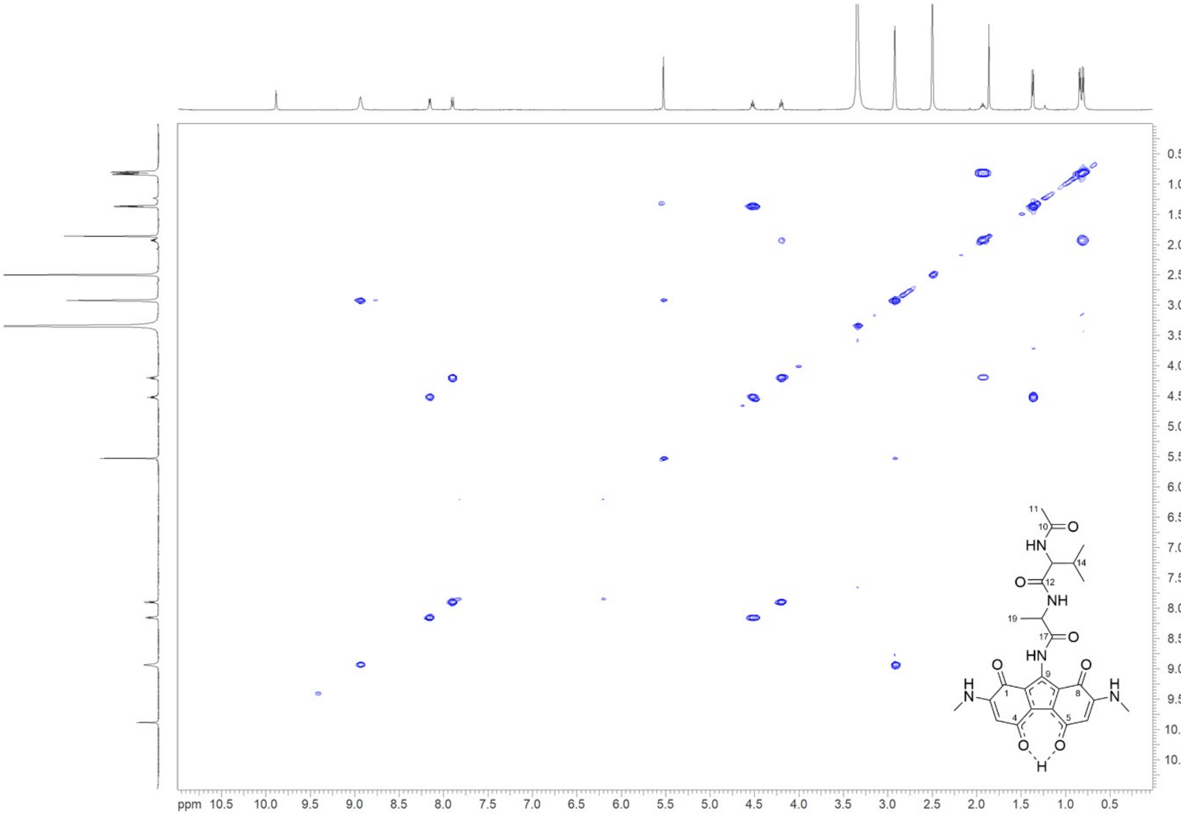

Figure S6: COSY spectrum (DMSO-*d*6) of malevonin.

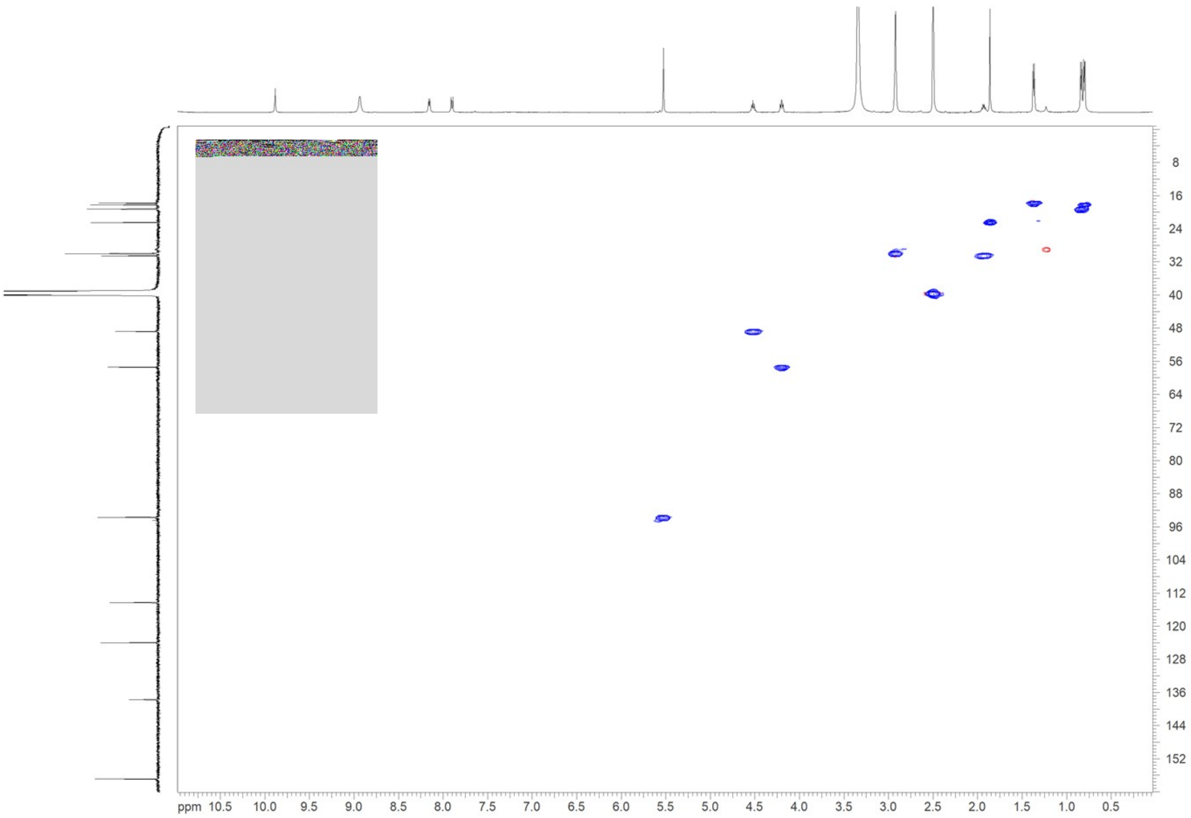

Figure S7: Edited HSQC spectrum (DMSO-*d*6) of malevonin.

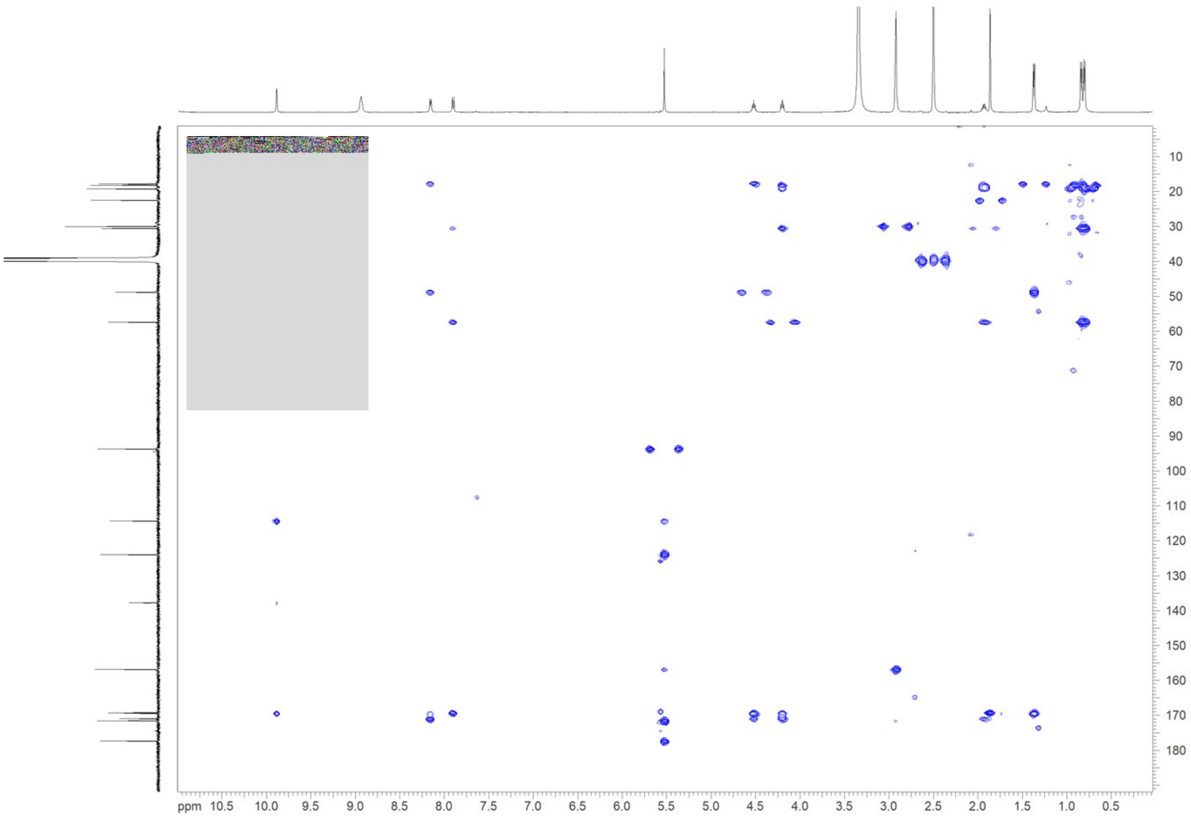

Figure S8: HMBC spectrum (DMSO-*d*6) of malevonin.

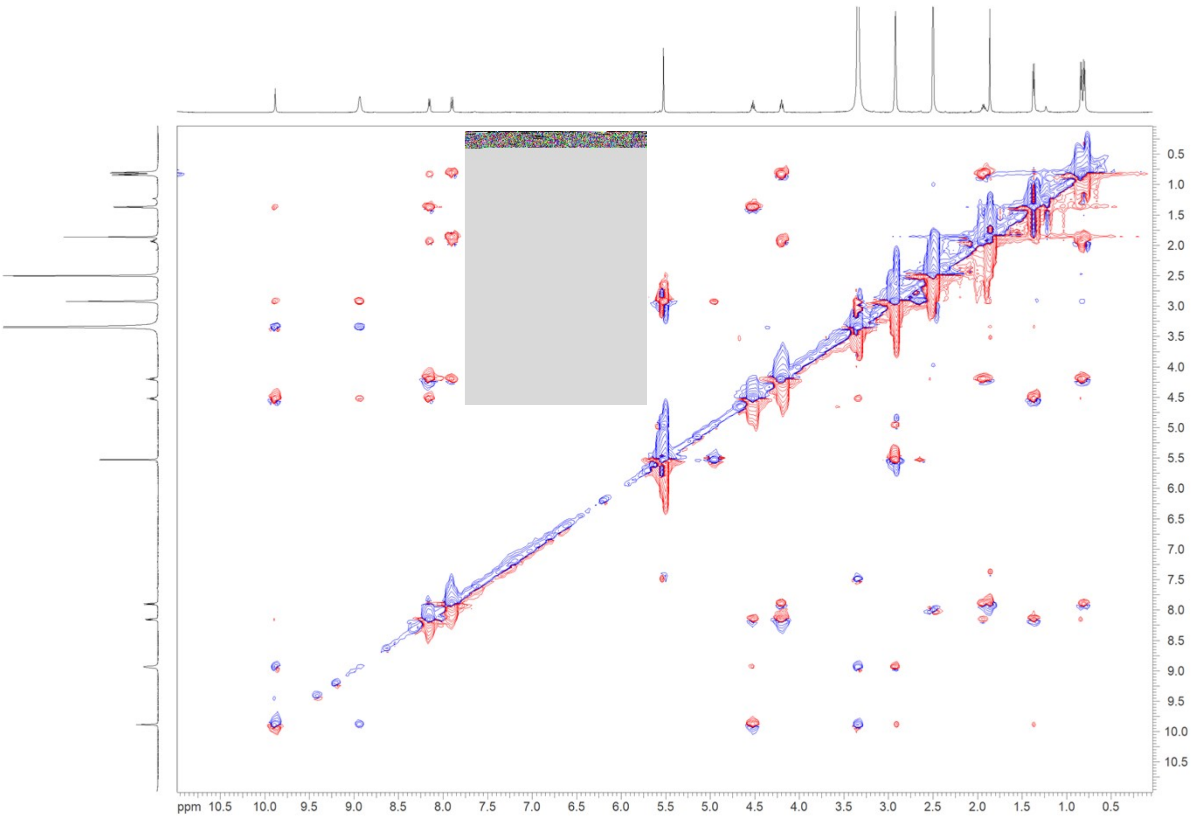

Figure S9: ROESY spectrum (DMSO-*d6*) of malevonin.

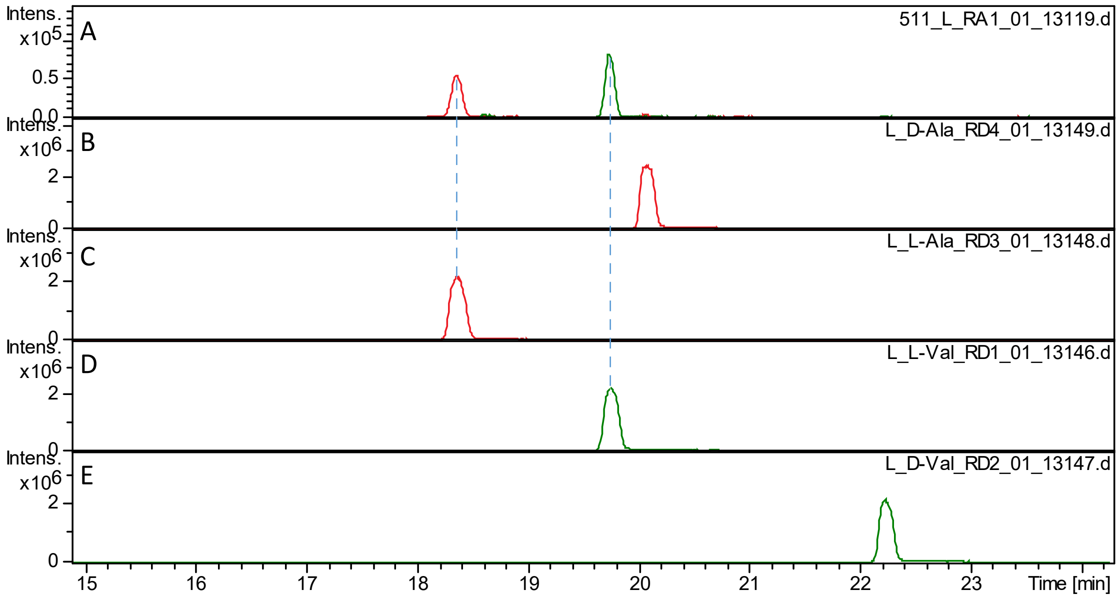

Figure S10: Determination of the absolute stereochemistry by Marfey's method using L-FDLA Derivatization agent with (**A**) malevonin hydrolyzed with 6M HCl and the standards (**B**, **C**) D/L-alanine and (**D**, **E**) L/D-valine.

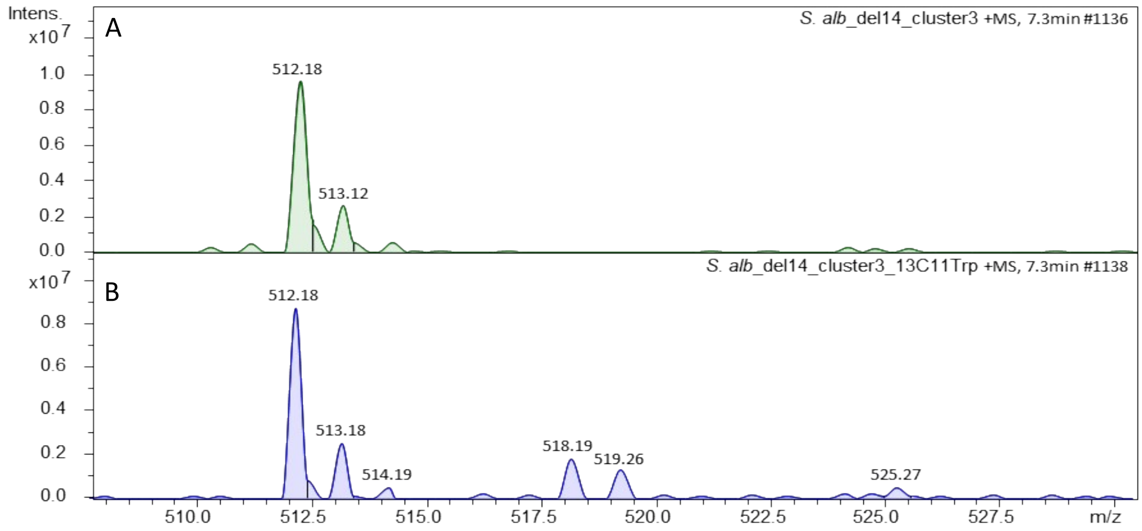

Figure S11: Feeding of *S. albus* Del14 with $^{13}C_{11}$ Trp (**2**) revealed (B) a mass of 518.18, 519.26 and 525.27 corresponding to +6, +7 and +13 Da compared to malevonin without labeling (A).

Score	Expect	Method	Identities	Positives	Gaps
699 bits(1805)	0.0	Compositional matrix adjust.	359/404(89%)	369/404(91%)	4/404(0%)

```
Orf24  1    SYGELDTTSTRLAYVLRTAFGVGPDAVAGVLLERGLDLPVAQLGVLKAGGAWLPLDPQHP  60
            SYGELD T+TRLA+VLR  FGVGPDAV GVLL+RGLDLPVAQLGVLKAGGAWLPLDPQHP
Orf25  1    SYGELDATATRLAHVLRADFGVGPDAVVGVLLDRGLDLPVAQLGVLKAGGAWLPLDPQHP  60

Orf24  61   AERLTGQLADAGARVVVTTRALATALPADMPRLCLDDPTTTHLLDRTPDTPLPHSGTKPE  120
             ERL   L+DA A  VVTTRALA ALPAD+PRL LDDP      L RTP+TPL    T P+
Orf25  61   TERLASYLSDARAAAVVTTRALAAALPADVPRLVLDDPAPRERLARTPETPL-TGNTDPD  119

Orf24  121  NLAYLIYTSGSTGAPKGVMVSHGAAVNFVLNARELFGIRPGDRLLQFANPAFDVSVFDFY  180
            +LAY+IYTSGSTGAPKGVMVSHGAAVNFVLNARELFGIRPGDRLLQFANPAFDVSVFDFY
Orf25  120  HLAYIIYTSGSTGAPKGVMVSHGAAVNFVLNARELFGIRPGDRLLQFANPAFDVSVFDFY  179

Orf24  181  GALGSGAAVVGASREVLLDPDALQELLVRERVSVAD---VPPAVLRLLDPGSLPDLRALF  237
            GALGSGAAVVGASRE LLDPDALQELLVRERVSVAD   VPPAVLRLLDPGSLPDLRALF
Orf25  180  GALGSGAAVVGASRETLLDPDALQELLVRERVSVADORFVPPAVLRLLDPGSLPDLRALF  239

Orf24  238  VGLEAFPAELVNRWSSEKREFHNGYGPTEATVACVDYLCPPGGLSASPPIGRAMANHRAY  297
            VGLEAFPAELVNRWSSEKREFHNGYGPTEATVACVDYLCPPGGLSASPPIGRAMANHRAY
Orf25  240  VGLEAFPAELVNRWSSEKREFHNGYGPTEATVACVDYLCPPGGLSASPPIGRAMANHRAY  299

Orf24  298  VLNAETFEPVPVGVPGELFVAGAGLARGYLNRPDLTAERFVPDPFSGSGERMYRTGDVVR  357
            VLNAETFEPVPVGVPGELFVAGAGLARGYLNRPDLTAERFVPDPFSGSGERMYRTGDVVR
Orf25  300  VLNAETFEPVPVGVPGELFVAGAGLARGYLNRPDLTAERFVPDPFSGSGERMYRTGDVVR  359

Orf24  358  WREDGNLEFLGRADRQVKIRGLRIEPGEIEHALAGCEGVRQGTV  401
            WREDGNLEFLGRADRQ+KIRGLRIEPGEIEHAL    GV Q  V
Orf25  360  WREDGNLEFLGRADRQIKIRGLRIEPGEIEHALTTSPGVAQAVV  403
```

Figure S12: Alignment of Orf24 and Orf25 from cluster 3 shows 90% similarity.

Table S7: Proposed functions of genes within cluster 16.

Orf	Proposed function	LocusTag in *S. alboniger* (CP023695.1)	GeneBank homologue
1	VOC family protein	CP975_33055	WP_150477657.1
2	NAD(P)/FAD-dependent oxidoreductase	CP975_33060	WP_150477658.1
3	CPBP family glutamic-type intramembrane protease	CP975_33065	WP_150477659.1
**4**	**helix-turn-helix domain-containing protein**	CP975_33070	WP_150478040.1
5	M64 family metallopeptidase	CP975_33075	WP_055536098.1
**6**	**LacI family DNA-binding transcriptional regulator**	CP975_33080	WP_150477660.1
7	nucleoside hydrolase	CP975_33085	WP_055536099.1
8	MFS transporter	CP975_33090	WP_055536100.1
9	terpene synthase family protein	CP975_33095	WP_055536101.1
10	family 2B encapsulin nanocompartment shell protein	CP975_33100	WP_055536102.1
11	hypothetical protein	CP975_33105	WP_055536103.1
12	MBL fold metallo-hydrolase	CP975_33110	WP_055536123.1
13	DUF4236 domain-containing protein	CP975_33115	WP_030775935.1
14	hypothetical protein	CP975_33120	WP_167532771.1
15	hypothetical protein	CP975_33125	WP_055536104.1
**17**	**AfsR/SARP family transcriptional regulator**	CP975_33130	WP_055536106.1
18	cytochrome P450	CP975_33135	WP_055536124.1
19	HAD family hydrolase	CP975_33140	WP_055536107.1
20	SigB/SigF/SigG family RNA polymerase sigma factor	CP975_33145	WP_055536108.1
21	aminotransferase class I/II-fold pyridoxal phosphate-dependent enzyme	CP975_33150	WP_055536109.1
22	hypothetical protein	CP975_33155	WP_246201713.1
23	PHP domain-containing protein	CP975_33160	WP_055536110.1
24	xanthine dehydrogenase family protein molybdopterin-binding subunit	CP975_33165	WP_055536111.1
25	FAD binding domain-containing protein	CP975_33170	WP_055536112.1
26	2Fe-2S iron-sulfur cluster-binding protein	CP975_33175	WP_055536113.1
27	hypothetical protein	CP975_33180	WP_055536114.1
28	alpha/beta hydrolase	CP975_33185	WP_055536115.1
29	ATP-binding protein	CP975_33190	WP_150477663.1
30	ribonuclease H family protein	CP975_33195	WP_055536116.1
**31**	**MarR family winged helix-turn-helix transcriptional regulator**	CP975_33200	WP_150478041.1
32	MFS transporter	CP975_33205	WP_055536118.1
33	iron-containing redox enzyme family protein	CP975_33210	WP_055536119.1
34	DUF6131 family protein	CP975_33215	WP_167532773.1
35	DUF5133 domain-containing protein	CP975_33220	WP_055536120.1
36	catalase	CP975_33225	WP_055536121.1
37	hypothetical protein	CP975_33230	WP_167532774.1

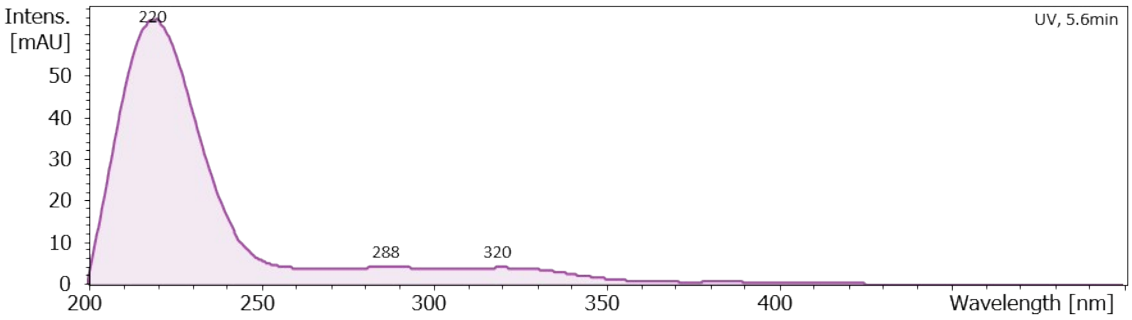

Figure S13: UV/VIS spectrum of mansevorone.

Table S8: NMR data (500 MHz, CD3OD) of mansevorone.

no	δ(^{13}C, ^{15}N) [ppm], type	δ(^{1}H) [ppm], mult (J)	COSY (H-)	HMBC (H-)	NOESY (H-)
1	143.8, CH	9.14, d (0.5)	4 (^{4}J)	1a, 3, 4, 4a, 5	
1a	150.5, C	-	-	-	
2-N	319.0, N	-	-	-	
3	153.8, C	-	-	-	
4	112.8, CH	8.23, d (0.5)	1 (^{4}J)	1a, 2-N, 3, 3', 5	2'
4a	130.5, C	-	-	-	
5	181.3, C	-	-	-	
6	112.8, CH	7.04, s	-	4a, 7, 7a	
7	165.7, C	-	-	-	
7a	162.2, C	-	-	-	
1'-NH	133.6, NH	-	-	-	
2'	127.1, CH	7.97, s	-	1'-NH, 3, 3', 8', 9'	4
3'	116.5, C	-	-	-	
4'	121.7, CH	8.30, m	5', 7' (^{4}J)	5', 8', 9'	
5'	121.7, CH	7.19, m	4', 6'	6', 7', 9'	
6'	123.4, CH	7.20, m	5', 7'	4', 8'	
7'	112.9, CH	7.46, m	6', 4' (^{4}J)	5', 9'	
8'	139.0, C	-	-	-	
9'	126.6, C	-	-	-	

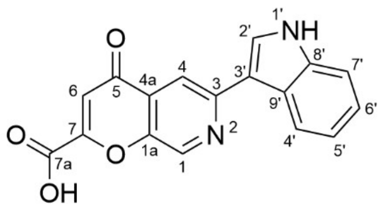

mansevorone (**2**)

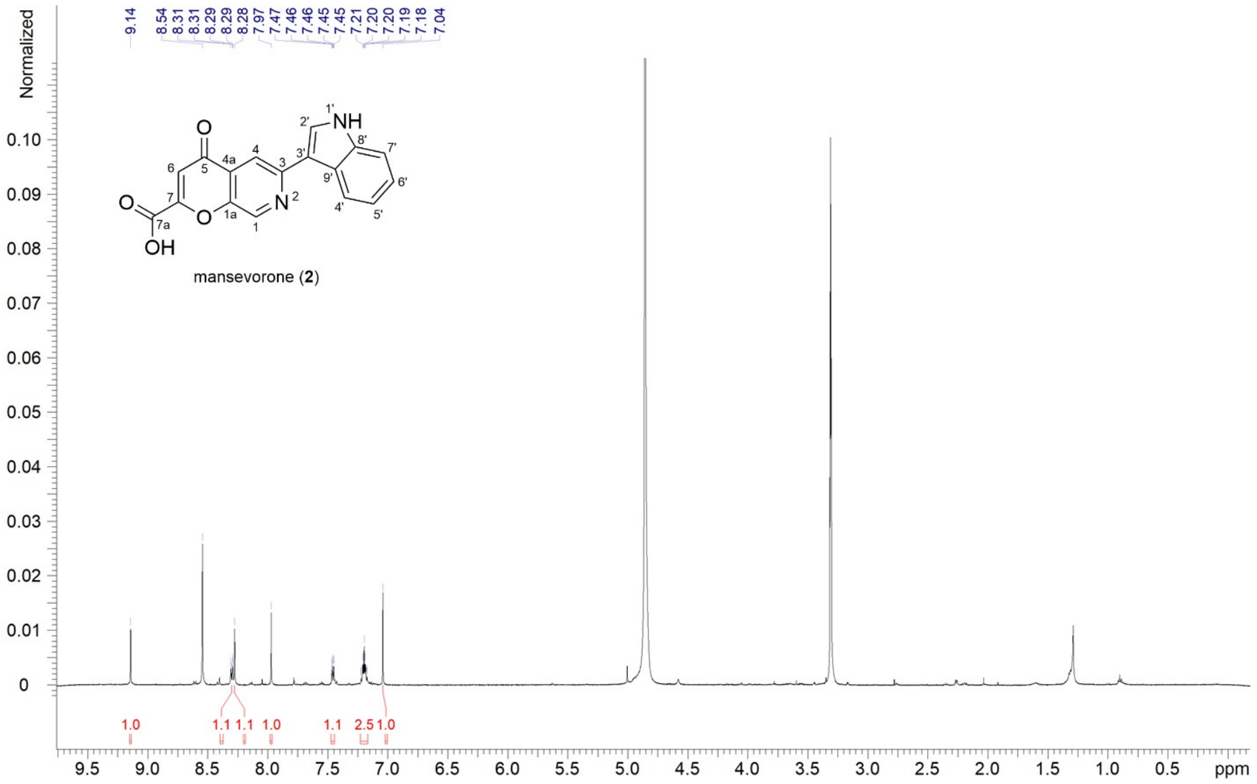

Figure S14. ^{1}H NMR spectrum (500 MHz, CD3OD) of mansevorone.

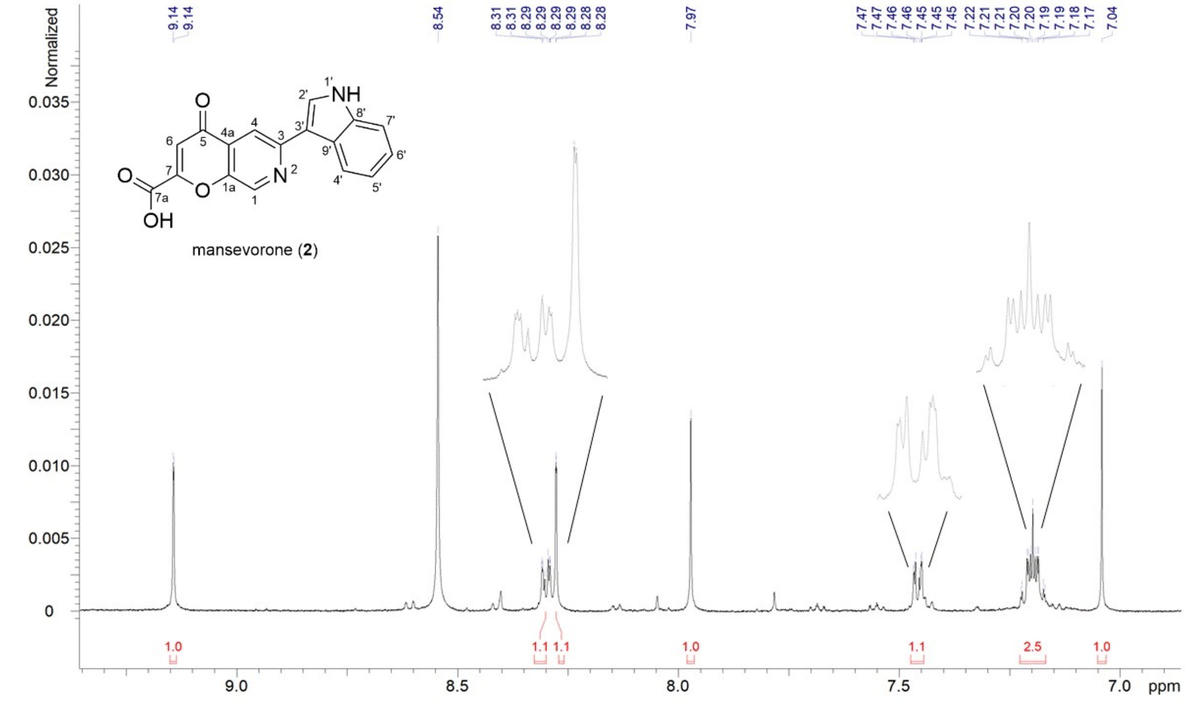

Figure S15. ^{1}H NMR spectrum (500 MHz, CD3OD) of mansevorone zoomed in on the relevant peaks.

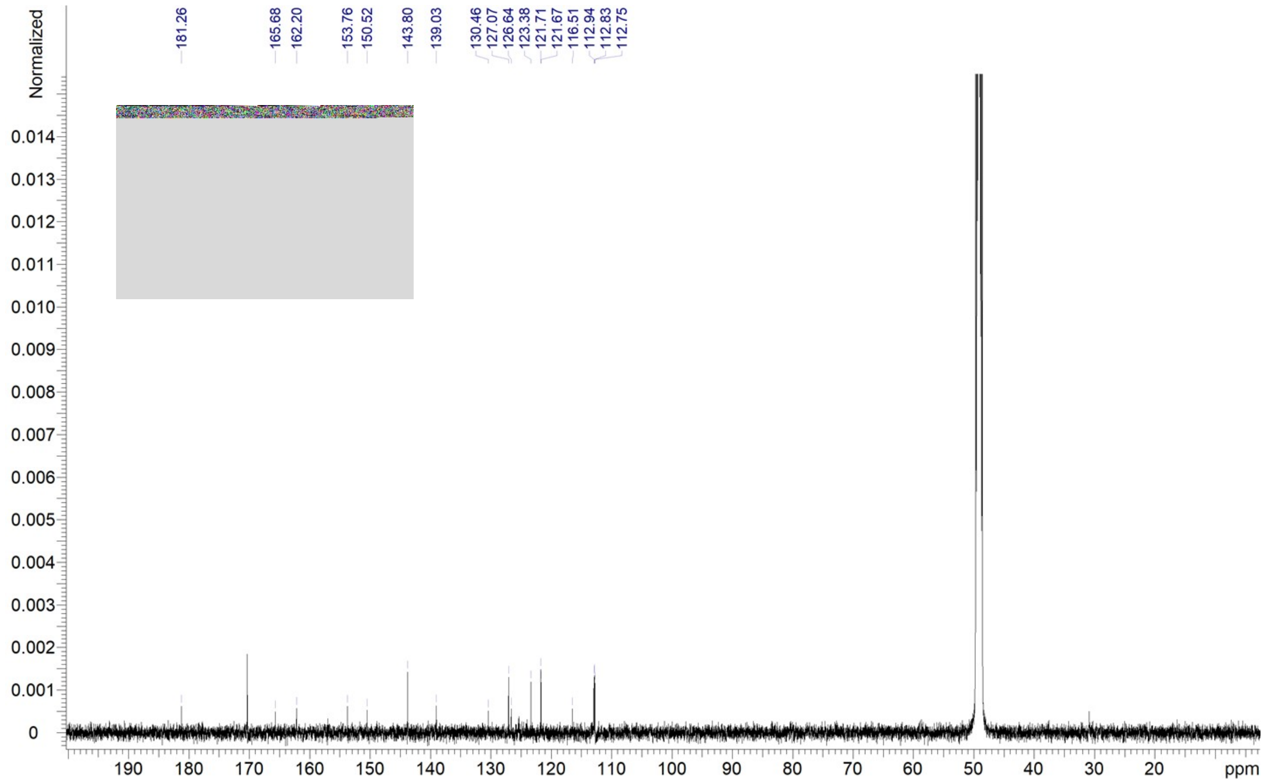

Figure S16: ^{13}C NMR spectrum (125MHz, CD3OD) of mansevorone

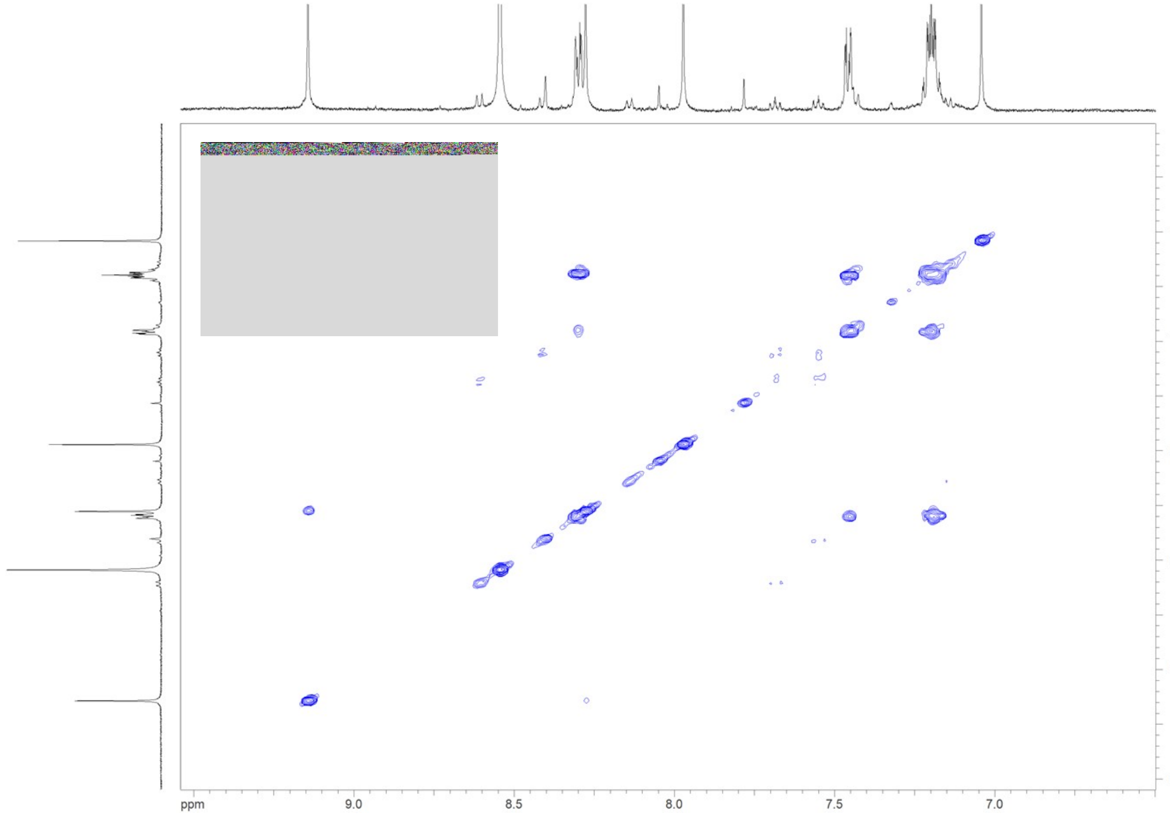

Figure S17: COSY spectrum (CD3OD) of mansevorone.

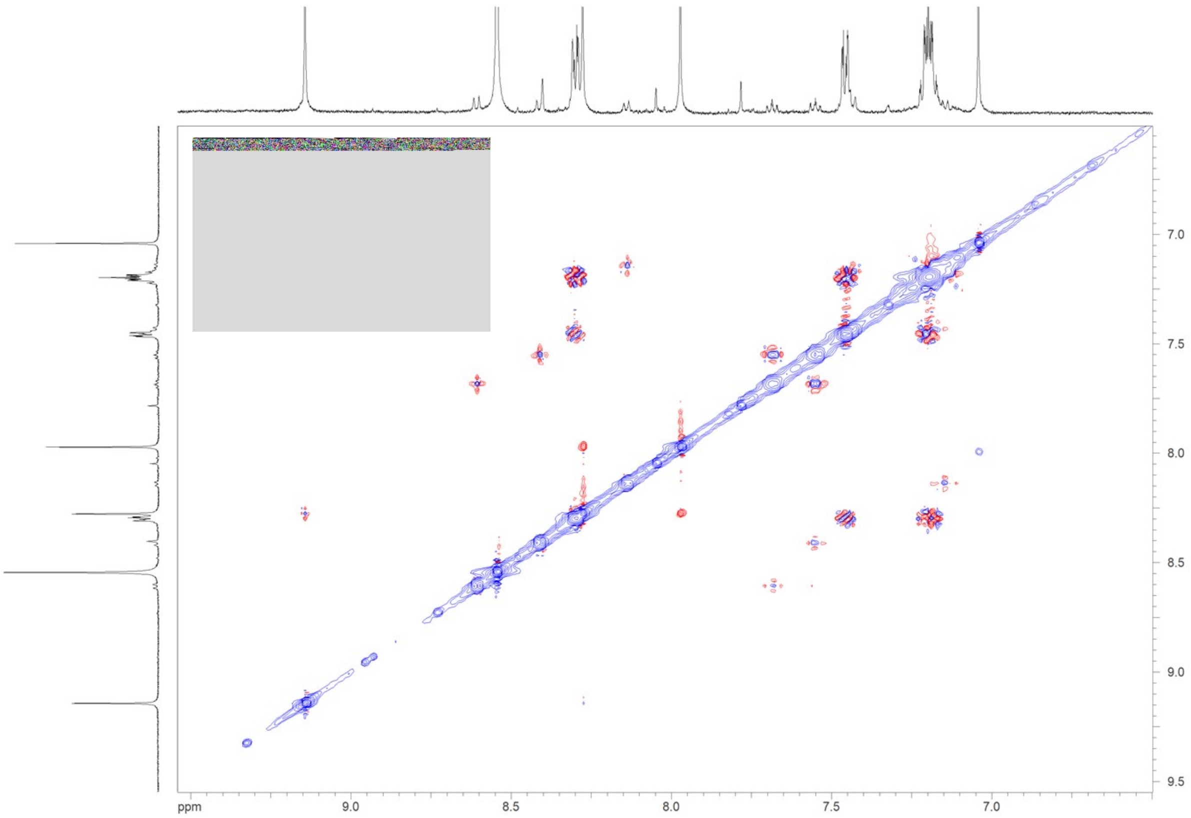

Figure S18: NOESY spectrum (CD3OD) of mansevorone.

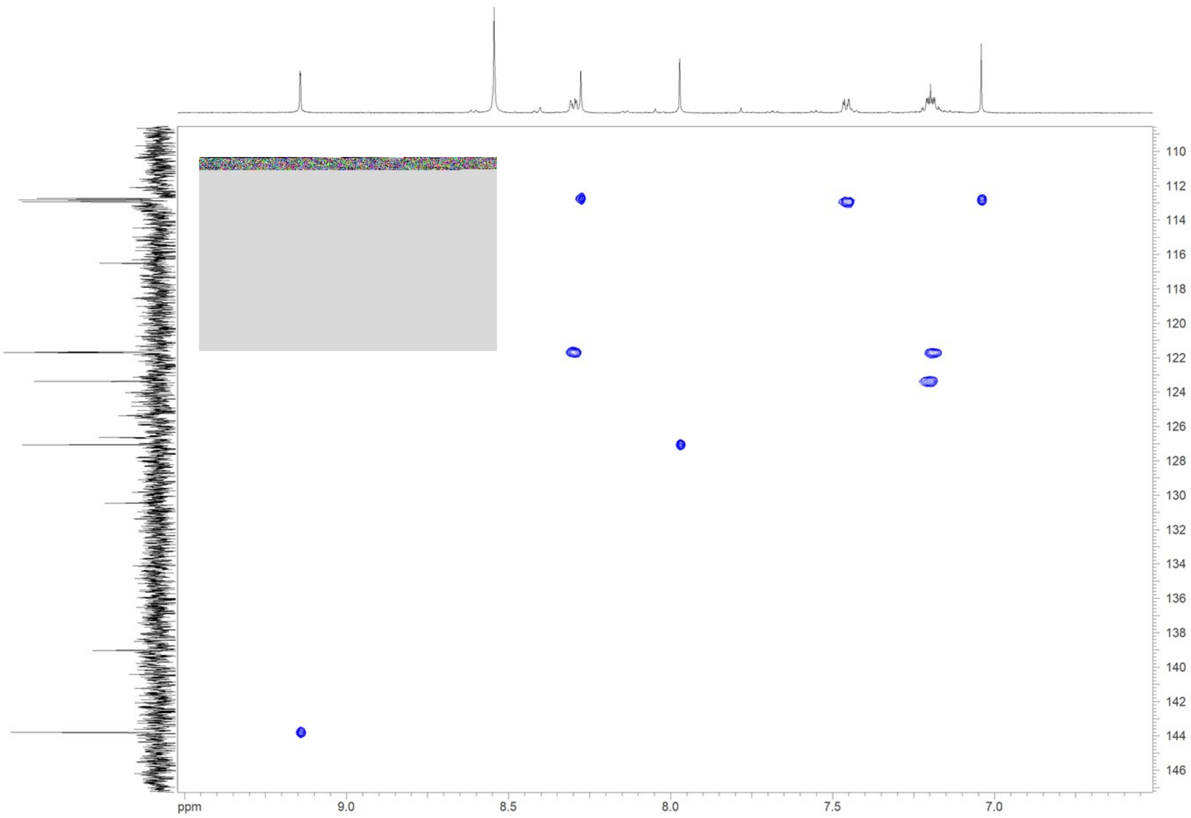

Figure S19: Edited HSQC spectrum (CD3OD) of mansevorone.

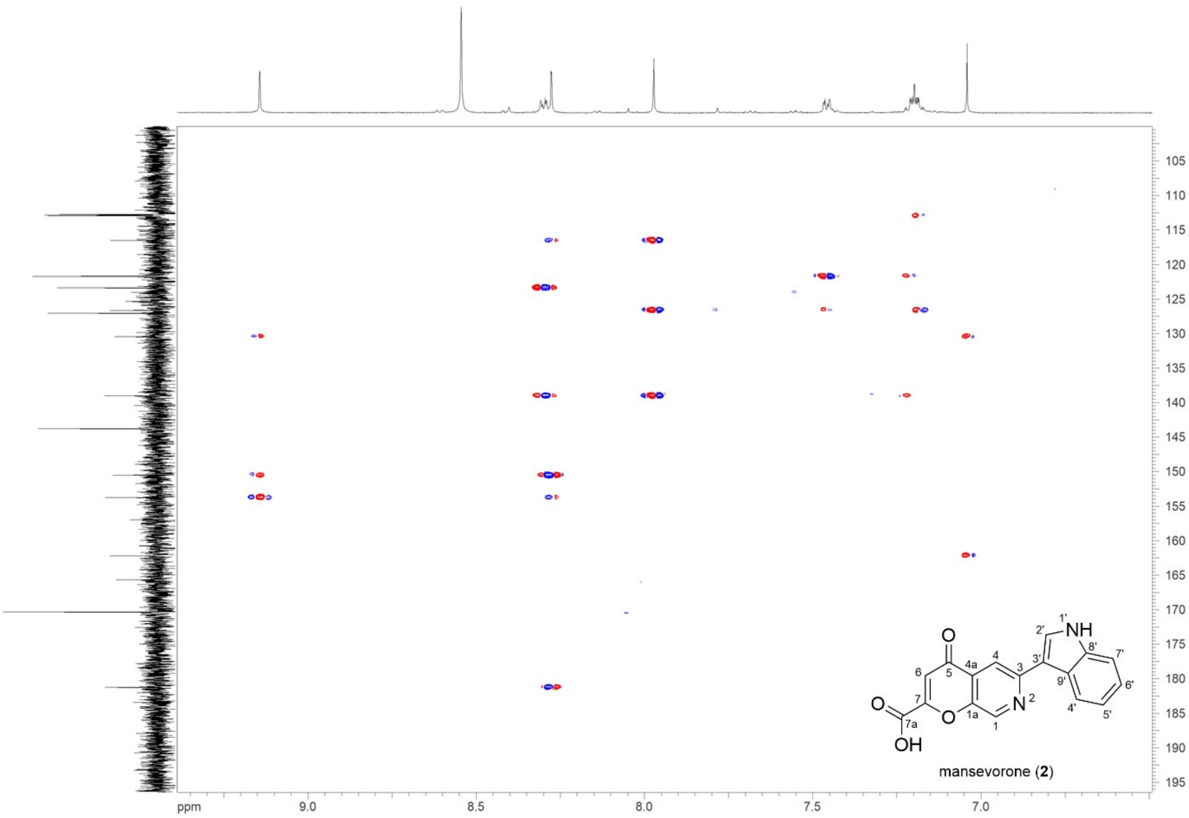

Figure S20: HMBC spectrum (CD3OD) of mansevorone.

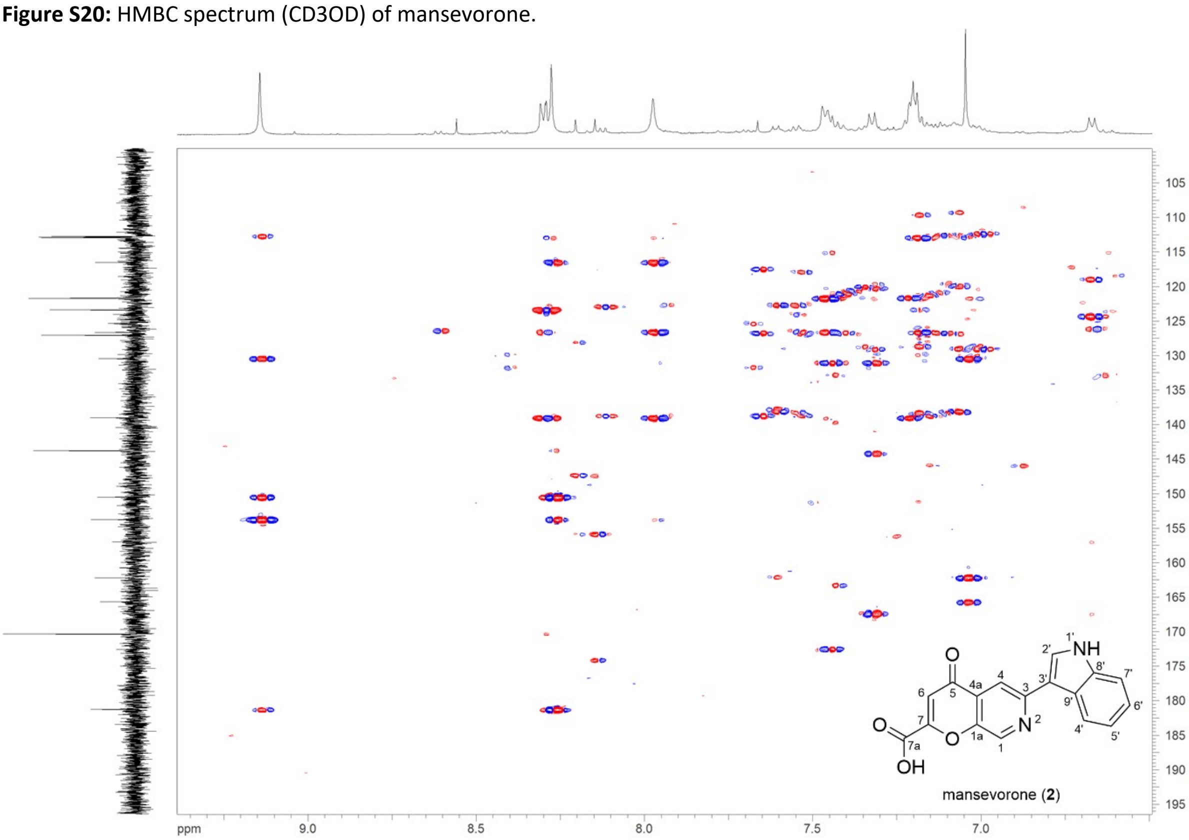

Figure S21: HMBC spectrum (CD3OD) of mansevorone before final HPLC purification.

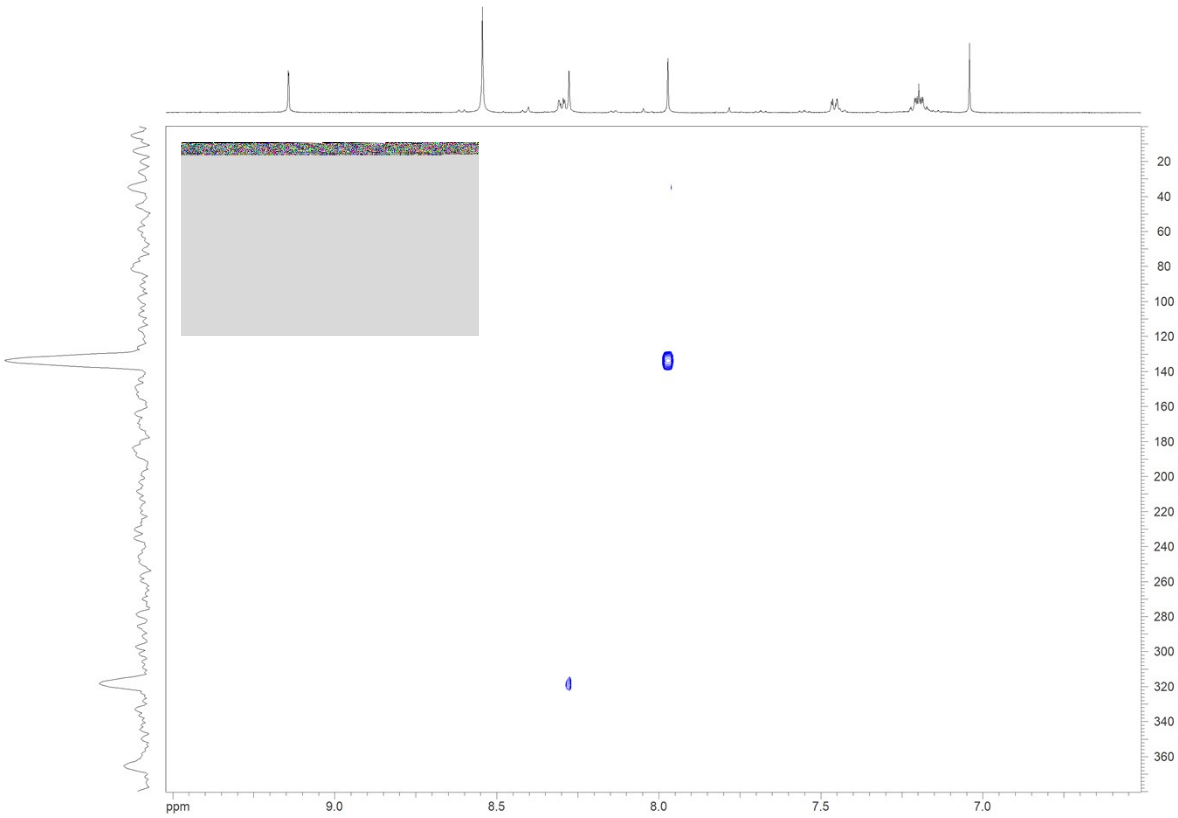

Figure S22: N-HMBC spectrum (CD3OD) of mansevorone.

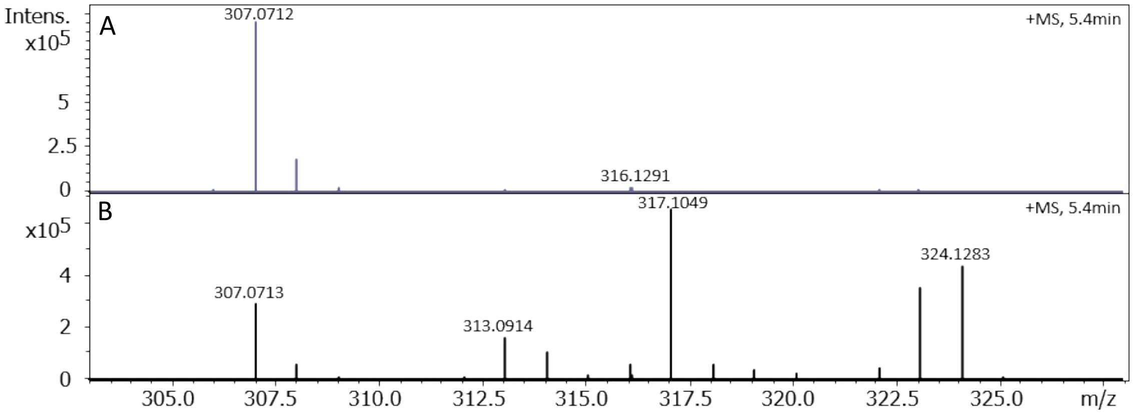

Figure S23: Comparison of MS spectra for mansevorone between (A) a culture of *S. albus* C16h7 and (B) a culture of *S. albus* C16h7 supplemented with $^{13}C_{11}$-labeled tryptophan.

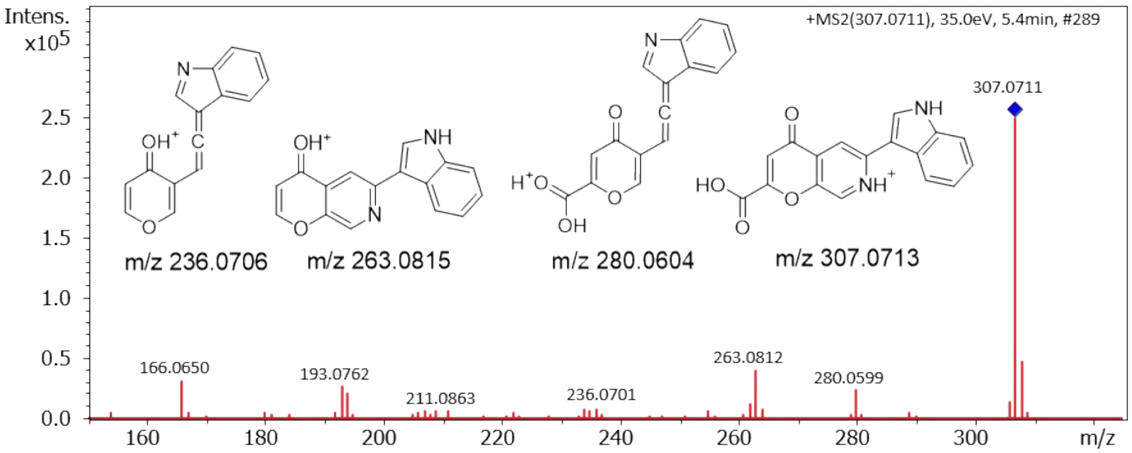

Figure S24*:* MS/MS fragmentation spectrum of mansevorone from cluster 16.

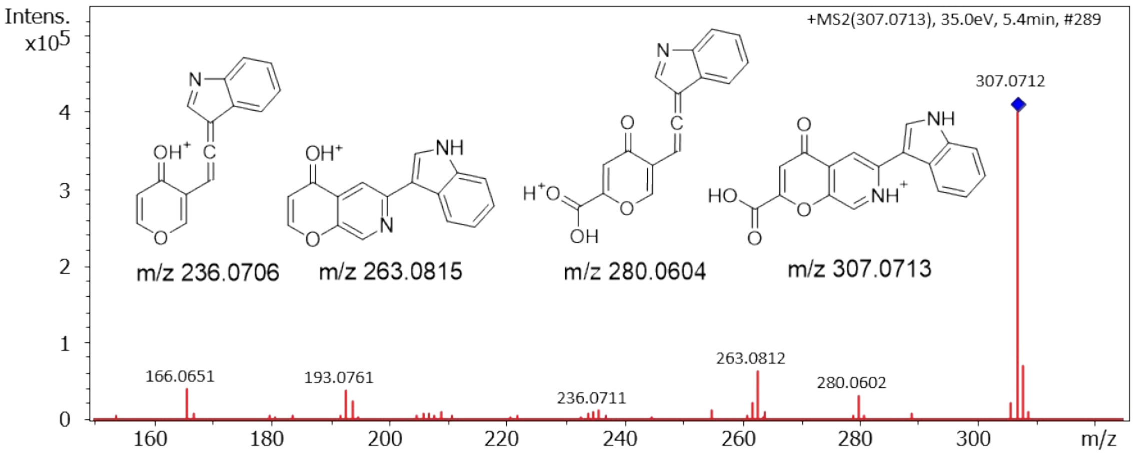

Figure S25: MS/MS fragmentation spectrum of mansevorone from cluster C1.8.

>AsfR sequence

MEFRLLGAVSVATEVGVLPLGPAKRRSLLAALLLRPNHPVPVDRLTAALWDQEPPARARGVIQGHVSQLRVLLTEAEAG
MFGVELVTQGTAYVLRMPESLLDAHRFEELVTLARGQRAPADAVAMYQEALSLWQGPALTGAYPSQPLQAAAQALEE
LRLASVESLAGAYTRMGEHARAAAVLRAEAGAHPLRESLSAELMRALQRAGRRTEALDWFHRTRRVLADELGVDPGRE
LADAYAAALRGAGDEADGAGGGASVADVPPSGGPVALAAPPASAPASLPAAPAAPGGSVVPAAPVASPASGPVLPPA
AIDLLPRMPRGFHGRAGELTALSRAAAGEAPVCLVTGPAGVGKTALVTHWAHRNREHFPGGLLYADLRGFSDTGEPAL
LEVLREFLLALGVAPRRIPESAGAASALFRSLSADRQLLVVLDNARASDQVRELLPGGARCVTVVTSRYRLRGLIASDAARP
VPVDVLEPEDSTALLAAVLGTDRVFAEEVAARRLAELCGGLPLALRVAAARLADQPESSLSTMCAELSDESRRLGLLDVED
TGVRAALRLTVRRLPADAARQFAHLGRHPGMYIDLYAAAALADTDPATAEAALDKLTAAHLVMRAGPDRWTLHDLVR
LYARDLDAEADALLRVLDLTVATALAAADAAEPGDESCFTLPADFRPPWAAREFADREQAMAWYAAERDDLTLAATA
ADSAGLHDRTWRIILGMWPQIVWRVQDGWAPLLKTALEATRADGDARAESRVLALLGWVLTEEGRIDEALGHLEAAP
LLAARAGDTRGEATALMNLSLAQAALGSPDEAAEGCALAAELAHSVGDRHTERLALCHLARHRLDAREWQAAHETAIA
ALDLVGPSEASAAARVLLLTAIGEALLGMGDETEGIRRLENAALEAEACGYDDGAVRALGALLRVSADAGLQARYDTAM
VRLMART*

Figure S26: Sequence of SARP regulator AsfR16 from cluster C16.

>DnrI sequence

MHALRTPGPSPERTDPAGPLLRVLGPMSARFDGQDLPLGPPRRRALLALLLIRLGRVVPTELIVDELWGDEPPRQPVATL
QSHVSHLRRVLDPTAGPGAASVLRYRAPGYVLRLAPEQVDFCRFEDLVSTGRRSLEQRDPLAARDRLAEALELWHGSPY
TEFSAHPPLADETARLEQVRLAALESYAEARLTLGAAAEVAADLDREVRNHPMRERLVGHLMTALTRLGRQAEALEVYE
RTRSHLVEEFGVDTAVELQRVRTAILRQELGDGGPGGATAAPAVSAPSAPLTPPARPTRSPASAVPAPYGGTGRPARTP
VPAPAAAPARAEDPGTDVPDPSAPWPFTGRDQELHRLAAAAAGALTGHGHVVSVLGPAGVGKTRLLMELAPRLETAD
EHLEVVWSHCFPGEGVPPYWLWTQVLRRLSATRPDAFRAATAPFGALLGPLMPERSAGPGGAPGPEVDWAQARFLT
HDAVCEVLLALAAESPLVLLLEDLHWADTASLDLLRLLGVRRLGHPLSIVLTARDFEIESDATMRRLLAEVVRGPRSETLRL
DGLPRQAVATLVEAQAGPGVGAEVVEALHRRSKGNPYFVMQLLSLLGDVRHLHDPGASAVLLAQVPTGVREALRQRF
SALPEPVLRVLRLCAVIGTEVDTDLLHRTADGDEPVAEALESAIRAGLLGEDPHHPGRLHFAHALVQETLAEEPAREERHR
LHARVAEALCARGRGQMGDEEIERVAHHSWHAKDALAPGETLPRLLRAAERAEHHLAYEQVETWLRRAVHLAGFLPA
DDPSAPGLEQRLHIQLGQVLATIRGYGDAEAEAALGRGRALSAVTHSPEDPSVLWALCAALLVTGRYDDSRQFSGLLRD
LAGRTRQPVAVLGAAYGEGIVLHVRGRLPEALAELEHGVDMADRFAREGHSLARTFQHDPRVSCRSYDTFTHWLLGDR
RTAAERRDQLLRLTDYESRPSDRAFALYVDAVVAAWEGDVGTALASGGEGARVAGEHGLLYWKAMLSLPAGWALTHS
GREEEGLALMRTSLDELCPSRTHLRLPLHLGLLGQAQFHAGRREEATDTLRRMLSVVERRREYVYLDPALPATRLLHELV
GREAAEAVLRGC*

Figure S27*:* Sequence of SARP regulator DnrI from cluster C1.8.

Score	Expect	Method	Identities	Positives	Gaps
144 bits(364)	4e-39	Compositional matrix adjust.	144/372(39%)	178/372(47%)	13/372(3%)

```
DnrI  21    LRVLGPMSARFDGQDLPLGPPRRRALLALLLIRLGRVVPTELIVDELWGDEPPRQPVATL  80
             R+LG +S   +   LPLGP +RR+LLA LL+R    VP + +   LW  EPP +    +
AsfR  3     FRLLGAVSVATEVGVLPLGPAKRRSLLAALLLRPNHPVPVDRLTAALWDQEPPARARGVI  62

DnrI  81    QSHVSHLRRVLDPTAGPGAASVLRYRAPGYVLRLAPEQVDFCRFEDLVSTGRRSLEQRDP  140
            Q HVS LR +L           L  +   YVLR+    +D  RFE+LV+  R    QR P
AsfR  63    QGHVSQLRVLLTEAEAGMFGVELVTQGTAYVLRMPESLLDAHRFEELVTLAR---GQRAP  119

DnrI  141   LAARDRLAEALELWHGSPYTEFSAHPPLADETARLEQVRLAALESYAEARLTLGAAAEVA  200
              A     EAL LW G   T      PL      LE++RLA++ES A A   +G  A  A
AsfR  120   ADAVAMYQEALSLWQGPALTGAYPSQPLQAAAQALEELRLASVESLAGAYTRMGEHARAA  179

DnrI  201   ADLDREVRNHPMRERLVGHLMTALTRLGRQAEALEVYERTRSHLVEEFGVDTAVELQRVR  260
            A L  E   HP+RE L   LM AL R GR+ EAL+ + RTR  L +E GVD   EL
AsfR  180   AVLRAEAGAHPLRESLSAELMRALQRAGRRTEALDWFHRTRRVLADELGVDPGRELADAY  239

DnrI  261   TAILRQELGDGGPGGATAAPAVSAPSA---PLTPPARPTRSPASAVPAPYGGTGRPARTP  317
             A LR    +    G  A+ A   PS     L  P     +   A PA  GG+  PA  P
AsfR  240   AAALRGAGDEADGAGGGASVADVPPSGGPVALAAPPASAPASLPAAPAAPGGSVVPA-AP  298

DnrI  318   VPAPAAAPARAEDPGTDVPDPSAPWPFTGRDQELHRLAAAAAGALTGHGHVVSVLGPAGV  377
            V +PA+ P     P      P  P  F GR  EL  L+ AAA    G   V  V GPAGV
AsfR  299   VASPASGPVL--PPAAIDLLPRMPRGFHGRAGELTALSRAAA----GEAPVCLVTGPAGV  352

DnrI  378   GKTRLLMELAPR  389
            GKT L+   A R
AsfR  353   GKTALVTHWAHR  364
```

Figure S28: Alignmet of SARP regulators DnrI from cluster 1.8 and AsfR from cluster 16.

Table S9: NMR data of 5'-Cl-Mansouramycin D (500MHz, CD3OD) and lit. data of mansouramycin D.

	5'-Cl-Mansouramycin D (CD3OD)				Mansouramycin D (DMSO-*d*6)**	
**No**	**δ(^{13}C) [ppm], type**	**δ(^{13}C) [ppm], predicted***	**δ(^{1}H) [ppm], mult. (J)**	**COSY (H-)**	**δ(^{13}C) [ppm]**	**δ(^{1}H) [ppm], mult. (J)**
1	147.9, CH	149.0	9.20, s		147.6	9.11, d (0.7)
3						
4	113.6, CH	114.6	8.22, s		113.0	8.18, d (0.7)
5						
6	98.9, CH	99.6	5.79, s	N-Me	99.4	5.71, d (0.4)
7						
7-NH						
N-Me	27.8, CH	29.4	2.95, s	6	28.6	2.82 d (5.0)
8						
9						
10						
1'-NH						
2'	129.6, CH	131.6	8.25, s		129.8	8.53, br s
3'						
4'	121.1, CH	119.07	8.55, s	6'	121.7	8.52, dd (1.9, 6.0)
5'					122.2	7.22, m
6'	122.7, CH	123.85	7.22, dd (2.0, 8.6)	7', 4'	120.8	7.20, m
7'	112.7, CH	113.13	7.45, d (8.5)	6'	111.9	7.48, m
8'						
9'						

* predicted by ACD Labs ** ACS Chem. Biol. 2022, 17, 3, 598–608

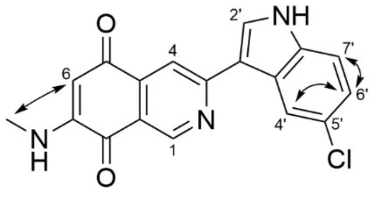

5'-Cl mansouramycin D (**3**)

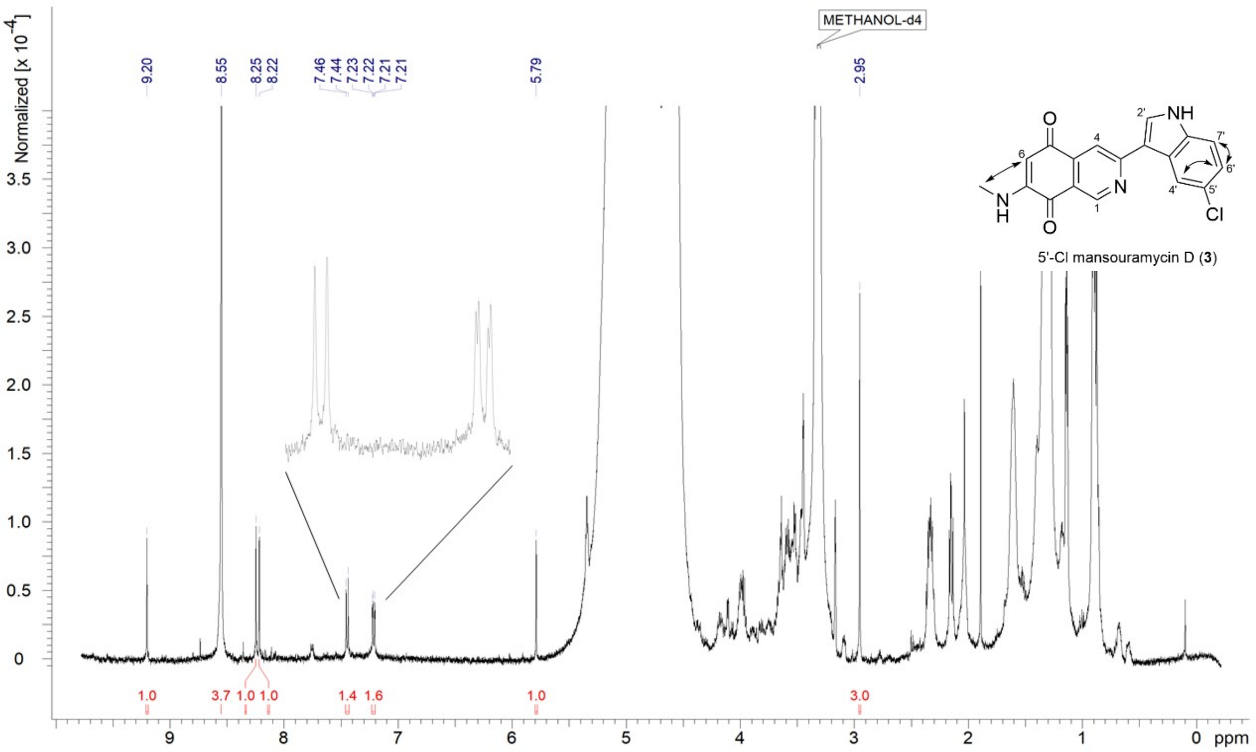

Figure S29: ^{1}H NMR spectrum (500 MHz, CD3OD) of 5'-Cl-mansouramycin D.

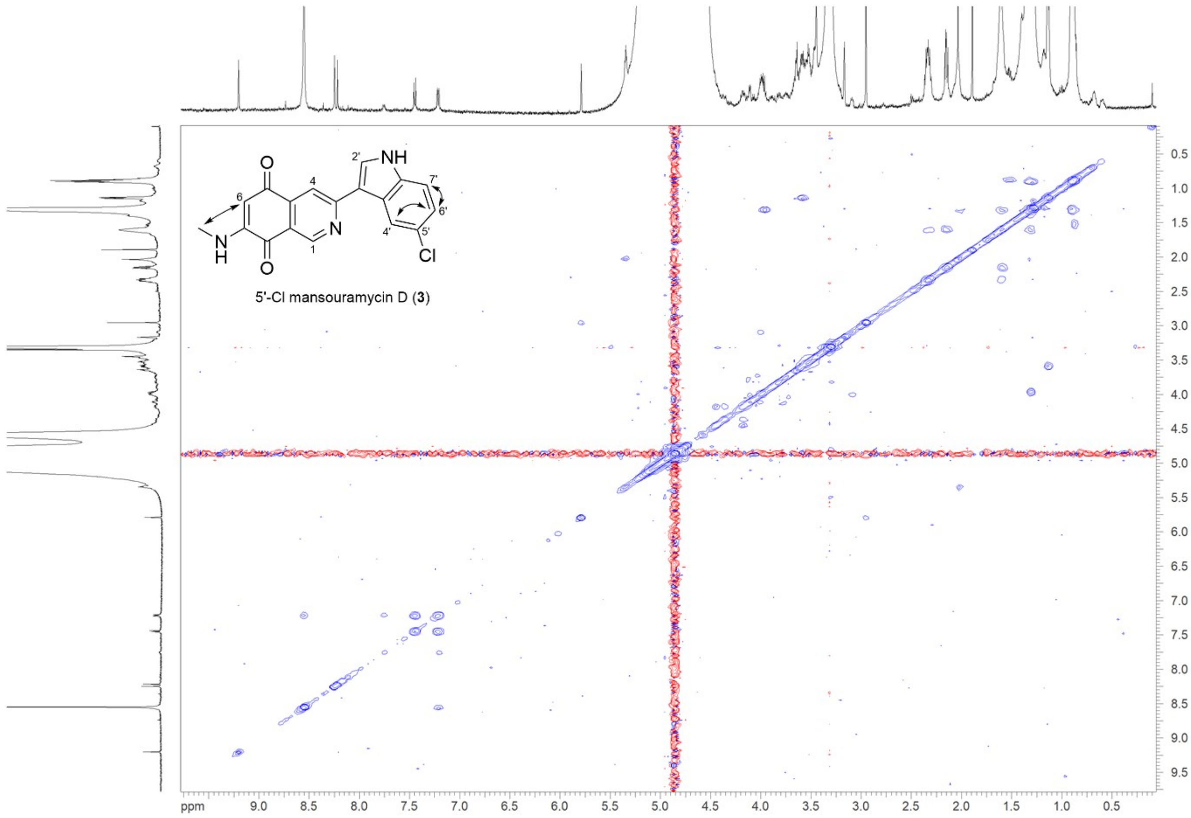

Figure S30: COSY spectrum (CD3OD) of 5'-Cl-mansouramycin D.

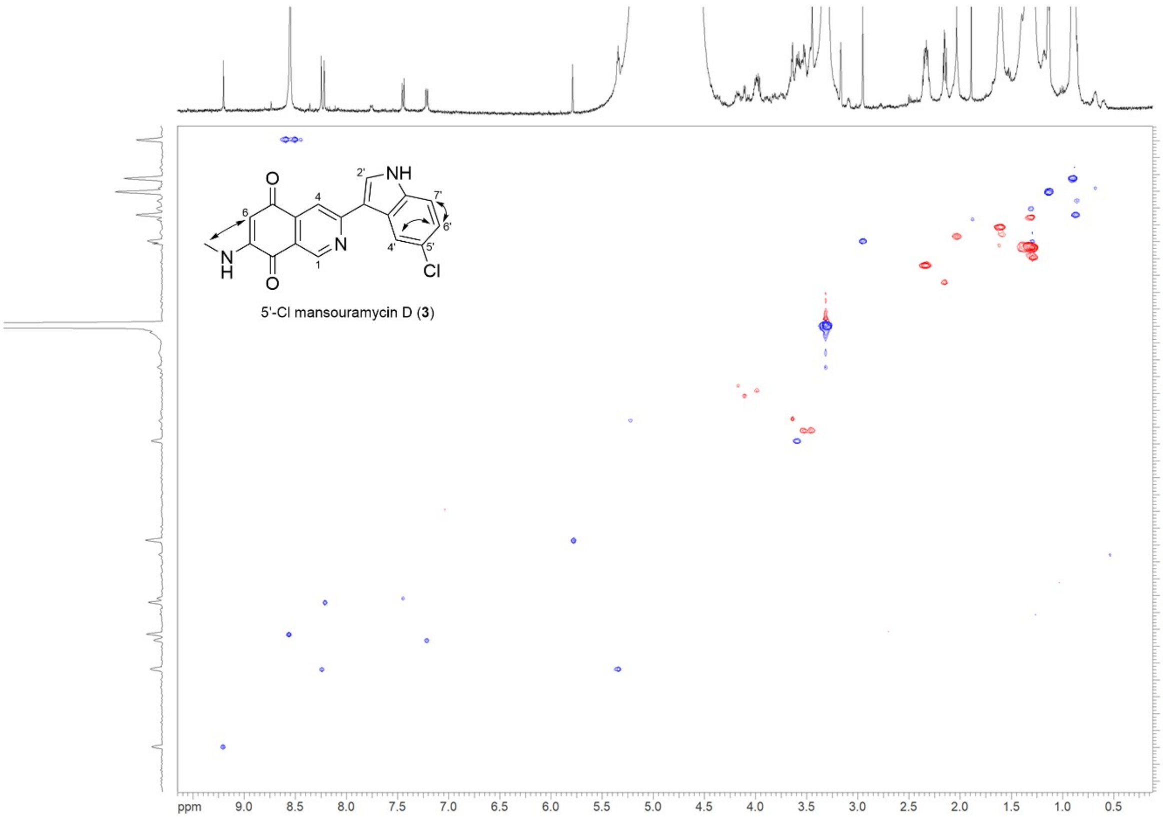

Figure S31: Edited HSQC spectrum (CD3OD) of 5'-Cl-mansouramycin D.

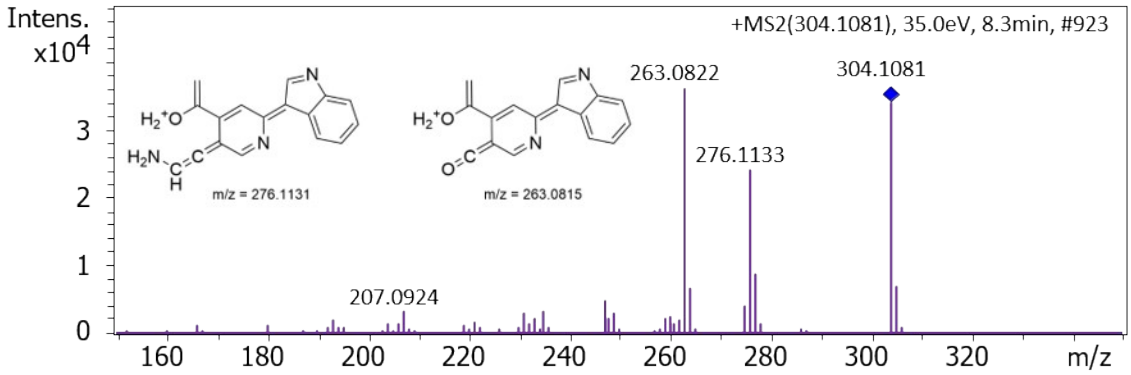

Figure S32: MS/MS fragmentation spectrum of mansouramycin D.

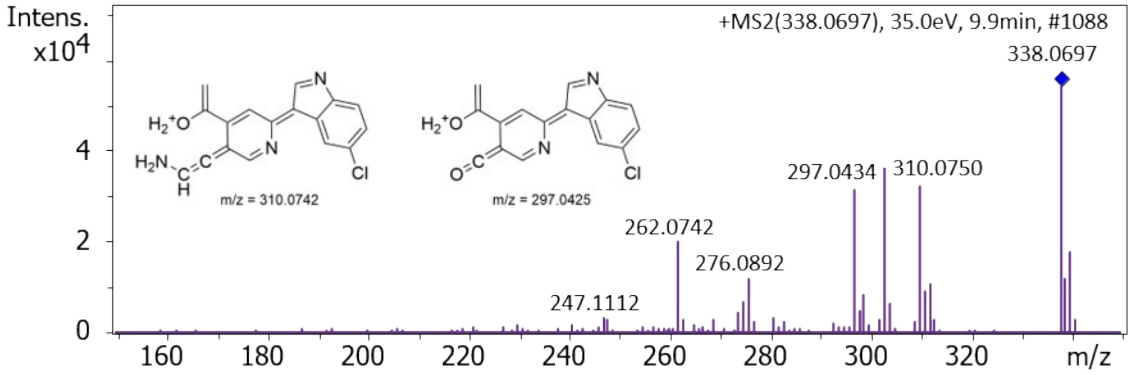

Figure S33: MS/MS fragmentation spectrum of 5’-Cl-mansouramycin D.

>RebH sequence

GTGATTAATTCAGTGGTCATTGTCGGCGGCGGCACTGCCGGATGGATGAGCGCCTCCTACCTCAAGGCGGCCTAC
GGTGACCGCATCAACGTCACTCTGGTGGAATCCGACCGCGTCGCGACCATCGGCGTCGGTGAAGCGACCTTCAGC
ACGGTCCGGCATTTCTTCGACTATCTCGGCCTGGACGAGCGCGAATGGATGCCGGAGTGCTCCGGTTCGTACAAGC
TGGGCATCCGGTTCGAGAACTGGCGGGAGCCGGGGCAGCACTTCTACCACCCCTTCGAGCGGCTGCGGACCTCCG
ACGGGTACACCCTGGCCGACTGGTGGCTTCAGGAGGGCGACCGCAGCGAGCCGTTCGACCGTTCCTGCTTCATCA
CGCCGGCGCTGTGCGAGGCCAAGCGCTCGCCGCGGCTGCTCGACGGCTCCCTGTTCGCGGGCGGTCTCGACGGCT
CGCTGGGCCGCTCGACGCTGGAGGAGCAGCGGTCCCAGTTCCCGTACGCGTACCACTTCGACGCGGCCCTGCTGG
CGAAGTTCCTGACCAAGTACGGCACCGACCGCGGCGTCCGCCATGTCGTCGACGACGTGACCGAGGTGGGCCGCG
ACGAGCGCGGCTGGATCAGCCATGTCGCCACCCGTGAGCACGGTGACCTCACCGGTGACCTGTTCATCGACTGCAC
CGGCTTCAAGGGCATGCTGATCAACGAGACCCTGGAGGAGCCGTTCGAGTCCTTCCAGGACGTGCTGCCGAACAA
CCGCGCCGTCGCGCTGCGCGTCCCGCAGGACGACCAGGCCACCACCGGGATGAACCCGTACACCACGGCGACCGC
GATGGACGCCGGCTGGATCTGGAACATCCCGCTGTTCGGGCGGAACGGCAACGGCTATGTGTACTCCGACGAGTT
CTGCTCCCCGGAGGAGGCGGAGCGCACCCTGCGGAACCACGTCGCCCCCGGCCGGGACGACCTGGAGGCCAACC
ACATCCGGATGCGCATCGGGCGCAACCGCCGCTCGTGGGTCAACAACTGTGTGGCCATCGGCCTGTCCAGCGCCTT
CGTCGAGCCGCTGGAGTCCACCGGCATCTTCTTCATCCAGCACGGCATCGAGCAGCTGGTGAAGAACTTCCCGGAC
GAGCGCTGGGACCCGGCCCTGGCCGACGACTACAACAACCGGGTCGCCGAGGTCCTGGACGGCGTCAAGGAGTT
CCTGGTCCTGCACTACAAGGCGGCGCAGCGCGAGGACACCCCGTACTGGAAGGAGGCCAAGACCCGTGCCCTGCC
CGACGGGCTCGCCGAGCGCCTCGCCATCGGCACCTCGCACCTGCTCGACGAGCGCACCATCTACCAGCCGTACCAC
GGCTTCGAGCAGTACTCCTGGATCACGATGATGCTGGGCCTCGGCCATGAGCCGGAGCGGCCGCGCCCCTCCCTC
GCGCACATCGACCCGACGAACGCGCGCGCCGAGCTCGCGCGGCTCCGGGCGGACGCGGACGAGCTGGTCGCCGC
CCTGCCCAGCTGCTACGAGTACATCGCCTCGCTCAACAGCTGA

Figure S34: DNA sequence of the tryptophan halogenase enzyme RebH.

References

1 Ahmed, Y.; Rebets, Y.; Estévez, M. R.; Zapp, J.; Myronovskyi, M.; Luzhetskyy, A., *Microbial cell factories* 2020, **19**, 1-16.
2 Myronovskyi, M.; Rosenkränzer, B.; Nadmid, S.; Pujic, P.; Normand, P.; Luzhetskyy, A., *Metabolic engineering* 2018, **49**, 316-324.
3 Shuai, H.; Myronovskyi, M.; Rosenkränzer, B.; Paulus, C.; Nadmid, S.; Stierhof, M.; Kolling, D.; Luzhetskyy, A., *ACS Chemical Biology* 2022, **17**, 598-608.
4 Flett, F.; Mersinias, V.; Smith, C. P., *FEMS microbiology letters* 1997, **155**, 223-229.
5 Grant, S. G.; Jessee, J.; Bloom, F. R.; Hanahan, D., *Proceedings of the National Academy of Sciences* 1990, **87**, 4645-4649.
6 Zhang, Y.; Muyrers, J. P.; Testa, G.; Stewart, A. F., *Nature biotechnology* 2000, **18**, 1314-1317.
7 Baker Brachmann, C.; Davies, A.; Cost, G. J.; Caputo, E.; Li, J.; Hieter, P.; Boeke, J. D., *Yeast* 1998, **14**, 115-132.
8 Bilyk, O.; Sekurova, O. N.; Zotchev, S. B.; Luzhetskyy, A., *PloS one* 2016, **11**, e0158682.
9 Oberhäuser, P.; Myronovskyi, M.; Stierhof, M.; Gromyko, O.; Luzhetskyy, A., *Microbial Cell Factories* 2025, **24**, 1-11.
10 Gregory, M. A.; Till, R.; Smith, M. C., *Journal of bacteriology* 2003, **185**, 5320-5323.
